# Robust 3D Position Estimation in Wide and Unconstrained Indoor Environments

**DOI:** 10.3390/s151229862

**Published:** 2015-12-14

**Authors:** Annette Mossel

**Affiliations:** Institute of Software Technology and Interactive Systems, Vienna University of Technology, Favoritenstr, 9–11/188/2, Vienna 1040, Austria; mossel@ims.tuwien.ac.at; Tel.: +43-1-58801-18893; Fax: +43-1-58801-918893

**Keywords:** indoor positioning, optical, wide-area, virtual teality, surveying

## Abstract

In this paper, a system for 3D position estimation in wide, unconstrained indoor environments is presented that employs infrared optical outside-in tracking of rigid-body targets with a stereo camera rig. To overcome limitations of state-of-the-art optical tracking systems, a pipeline for robust target identification and 3D point reconstruction has been investigated that enables camera calibration and tracking in environments with poor illumination, static and moving ambient light sources, occlusions and harsh conditions, such as fog. For evaluation, the system has been successfully applied in three different wide and unconstrained indoor environments, (1) user tracking for virtual and augmented reality applications, (2) handheld target tracking for tunneling and (3) machine guidance for mining. The results of each use case are discussed to embed the presented approach into a larger technological and application context. The experimental results demonstrate the system’s capabilities to track targets up to 100 m. Comparing the proposed approach to prior art in optical tracking in terms of range coverage and accuracy, it significantly extends the available tracking range, while only requiring two cameras and providing a relative 3D point accuracy with sub-centimeter deviation up to 30 m and low-centimeter deviation up to 100 m.

## 1. Introduction

An increasing number of application areas require spatial context awareness in indoor environments. These areas comprise navigation, security and surveillance, virtual and augmented reality (VR/AR) and entertainment as well as remote control, *i.e.*, for underground construction tasks. As fundamental base for spatial context awareness, accurate position estimation with low latency of an object in 3D space—such as the user—is required. This position estimation is also referred to localization or 3 degree of freedom (DOF) tracking. However, due to the lack of availability of global positioning system (GPS) signals inside buildings, reliable and accurate 3DOF tracking remains a challenging topic. This is especially true when aiming at accurate position estimates at high update rates for wide-area tracking in unconstrained indoor environments.

A large number of different tracking technologies for indoor environments exist and each method has its advantages and disadvantages regarding volume coverage, tracking accuracy, sensitivity to interferences as well as scalability. Thus, there is no general tracking technology that perfectly suits all variations of tracking scenarios. Infrared optical tracking detects targets within camera images in the near infrared spectrum and has been found to be fast, accurate as well as scalable to a certain extent. It is less susceptible to noise compared to competing approaches, allows for simultaneous tracking of multiple objects, trackable optical markers can be individually designed, they are lightweight, re-configurable and wireless. However, state-of-the-art systems suffer from sensitivity to ambient static or moving light sources during calibration and tracking and only cover standard room sized environments with a small amount of vision hardware. This yields lack of tracking support for wide unconstrained indoor environments and results in high hardware costs and complex setup as well as maintenance routines when extending the tracking volume. Nowadays, optical tracking is widely used for position estimation in indoor localization, fostered by the fact that the majority of these setups are used—up till now—in controlled indoor environments. Thus, employing optical tracking to other application domains that are located in wide-area unconstrained indoor environments is impeded by the following three limitations: (1) Tracking coverage, (2) system sensitivity, and (3) costs.

### 1.1. Contribution

In this paper, an optical position tracking system is presented that features low latency position estimation, enhanced robustness against environmental interferences and extended volume coverage. Sub-parts of the presented system has been previously tested in several heterogeneous use cases, user tracking for VR/AR reality applications [1], handheld target tracking for tunneling [2] and machine guidance for mining [3]. The main contributions of this paper are:

1. Providing a detailed overview of the entire system and its capabilities across the use cases.
2. Extending and comparing the results from the heterogeneous use cases to embed the presented approach into a larger technological and application context.

**Figure 1:**
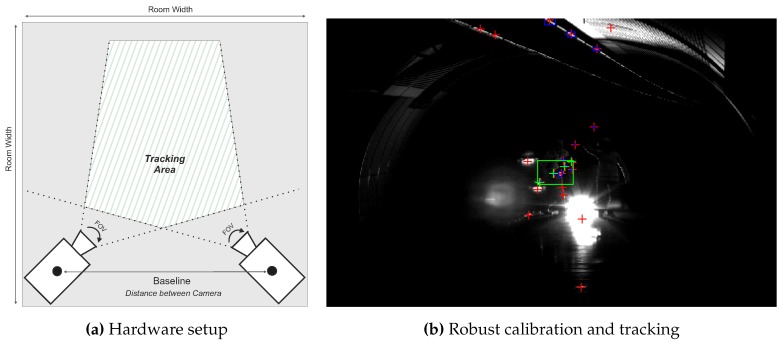
Key properties of the presented optical tracking system.

In Figure 1, the major properties and capabilities of the proposed system are shown. Figure 1a illustrates the system’s hardware setup while Figure 1b depicts a successfully detected target of our system in the camera image that can be subsequently employed for calibration and tracking. In summary, the presented system provides the following major capabilities:

- Wide-Area Tracking in Unconstrained Environments: The system provides model-based infrared optical tracking of multiple rigid-body targets in wide, unconstrained volumes while requiring a minimal hardware setup of two cameras. The system automatically tracks visible target(s), thus temporary occlusions can be recovered and no initial manual sighting is necessary as it is a prerequisite of laser measurement systems. At each stage of the system’s workflow—target training, extrinsic camera calibration and 3D position tracking—no constraining of the tracking volume is necessary. This enables the system to fully function in indoor environments with static and moving light sources, varying ambient up to very poor illumination.
- Robust Camera Calibration: The presented external calibration approach allows for parameter estimation of stereo cameras rigs with wide baselines under varying illumination. Therefore, the tracking target is re-used to artificially generate point features that are crucial in poorly illuminated environments or in scenarios with little geometric structure. Furthermore, the target’s properties support reliable correspondence matching without requiring the epipolar geometry for correspondence analysis.
- Adaptable Design and Ease of Use; Targets are designed to be highly reconfigurable and are equipped with standard infrared light emitting diodes. They can act as a hand-held point measure unit or can be attached to arbitrary objects. The small amount of hardware enables quick system installation, yielding only minimal disturbances of activities within the tracking volume. No further preconditioning of the environment is necessary, increasing the system’s ease of use during setup and maintenance and making it unobtrusive and cost efficient.
- General Purpose Tracking System: The system’s design allow for application to a broad range of scenarios. To demonstrate the system’s capabilities to act a general purpose measurement tool in indoor environments, it was experimentally applied to three different unconstrained wide area scenarios. (1) position tracking for VR/AR tasks; (2) tunneling surveying tasks and (3) autonomous machine guidance for underground construction. The experimental results show relative millimeter point accuracy up to 30 m and centimeter deviation up to 90 m. These results clearly improve state-of-the-art systems and reveal the system’s applicability for a broad range of use cases.

With its features, the presented system addresses the need of cost-efficient, wide-area, robust, accurate 3D position estimation in indoor environments.

### 1.2. Related Work

To achieve the major objective of this work, to track objects in large, unconstrained indoor environments, the tracking system must be capable to cope with ambient interfering lights, infrared radiation, temporary occlusions and even harsh environmental conditions, such as fog and dust. In addition, the authors aim at tracking at large distances with a small amount of hardware to minimize the necessary preconditioning of the tracking environment. To track objects in space and especially in large volumes, different techniques exist from commercially available products to on-going research prototypes. Extensive research has been performed to develop indoor location systems (ILS) for enabling context-aware applications, user tracking and surveillance [4]. Since this work focuses on positioning in indoor environments, we do not discuss related work based on global navigation satellite systems (GNSS) or tracking solely based on inertial sensors, as inertial measurements suffer from significant drift over time, especially for position estimation. Moreover, we do not incorporate magnetic tracking into the discussion of related technologies, as it is subject to interference from ferromagnetic materials in the tracking volume and magnetic fields generated by other electronic devices, and it is sensitive against conductive materials that are placed near to emitters or sensors. These factors tremendously limit potential tracking environments and making it impractical for the intended environments. Regarding optical tracking, techniques based on natural features are not reviewed as well, since they require prominent and distinctive structures for pose estimation. These distinct features must either be found on the tracked object in an *Outside-In* scenario, or have to be distributed throughout the volume in an *Inside-Out* tracking setup [5]. For both scenarios, a reliable feature distribution and an adequate illumination cannot be guaranteed in all of the intended tracking environments that have been investigated and can be targeted with this work.

To summarize, the most relevant tracking technologies for the intended wide area indoor environments are radio frequency (RF), ultra-sonic and model-based optical systems. Since they all have advantages and disadvantages regarding accuracy, latency, reliability, scalability and cost, no de-facto standard has been established yet. Thus, we outline state-of-the-art ILS techniques and discuss their advantages and disadvantages.

#### 1.2.1. Radio Frequency and Ultra Sound

Radio frequency systems based on Wi-Fi infrastructure or radio-frequency identification (RFID) [6] require a number of readers within the measurement volume to enable object tracking with low latency in large volumes [7]. However, WiFi signals tend to be extremely noisy and signal strength highly depends on surrounding building structures and materials. Thus, precise position estimation cannot be guaranteed even with multiple readers in the volume. In addition, the extensive pre-conditioning of the tracking volume is cost-intensive due to the amount of necessary hardware. Recently, a number of commercially available ILS applications such as Google Indoor Maps [8], SensionLab [9] as well as Indoors [10] emerged to localize a smartphone (and thus its user) by fusing mobile cellular data, WiFi and inertial measurements to minimize position jitter from WiFi data. Google Indoor Maps optimizes the position accuracy by pre-measuring and mapping the signal strength of the WiFi spot within the volume. However, this process takes time before the actual tracking can start. Furthermore, all systems require pre-built indoor floor plans for position visualization and only provide—in best case—several meter accuracy.

Ultra-sonic location systems such as [11,12] rely on time-of-flight measurement of ultra-sonic signals, calculated using the velocity of sound. Such systems are scalable and can track multiple moving objects. However, current systems offer in the very best case meter-level accuracy under optimal conditions for 3D position estimation [13]. Furthermore, precision and range are not reliable since velocity of sound in the air is highly dependent on environmental conditions, especially humidity and temperature. In particular at long ranges, ultra-sonic systems are often extremely noisy and for that reason not a proper solution for our system’s objectives.

Compared to ultrasound, the RF-based Ultra Wide Band (UWB) technology enables distance measurements without line-of-sight requirements. An example for such a system is Ubisense [14] that employs TDoA (Time-Difference-of-Arrival) and AoA (Angle-of-Arrival) measurements between mobile tags and a minimum of four fixed base stations. It offers fast signal speed and hence high sample rates (approximately 135 Hz) and provides an accuracy of down to 0.2 m. The LPM system by Abatec [15] offers a sample rate of 1 kHz with an accuracy down to 0.10 m. It measures the distance between fixed base stations and mobile tags based on the frequency modulated continuous wave principle [16]. Although large distances can be covered, the ultrasound and RF-based systems are expensive and the resulting accuracy is not sufficient for precise user tracking for virtual reality applications.

#### 1.2.2. Optical Tracking

Model-based optical tracking systems require the target to be within the line-of-sight of one or more cameras to estimate its 3D coordinates from the 2D image-projections [17]. It is robust against magnetic, electric and acoustic interference and works with light-emitting (active) or retro-reflective (passive) targets. One camera is sufficient for tracking in an *Inside-Out* scenario; such as setup, *i.e.*, the InterSense IS 1200 system [18] is employing. It offers a scalable, cost-effective solution for wide area tracking as it fuses optical tracking of planar bitmap patterns [19,20] with inertial measurement data. Therefore, an inertial measurement unit is combined with a single camera and attached to the trackable object to observe passive markers that have to be distributed throughout the volume. While this setup offers high updates rates with very low latency (max. 8 ms) it requires sufficient illumination and a large number of targets that have to additionally be in close range to the camera to ensure robust tracking. These prerequisites make this system impractical and even impossible to apply for our intended environments. As the implicit nature of Inside-Out tracking requires well-distributed visual features throughout the volume, it can be concluded that using active targets would also not be a sufficient approach since it would violate the our goals of omitting pre-conditioning of the environment and of minimizing the necessary amount of hardware components.

*Outside-In* optical tracking systems require the target to be within the line-of-sight of two or multiple cameras. In the following, a number of state-of-the-art Outside-In model-based tracking systems are presented. The near infrared (NIR) spectrum based systems, such as Vicon [21], A.R.T [22] or iotracker [23,24] offer (sub)-millimeter accuracy in standard room sized environments (4×4×3 m${}^{3}$) and provide tracking of multiple targets with very low latency. To enlarge the tracking volume, those systems increase the number of employed cameras (up to 50 in A.R.T). However, this causes a tremendous growth of costs and setup complexity. The PPT-E system [25] is able to cover areas up to 20×20 m${}^{2}$ with a minimum of four cameras but sub-millimeter tracking accuracy is guaranteed only for volumes up to 3×3×3 m${}^{3}$. No accuracies are provided for larger volumes. The Prime41 system [26] offers multiple user tracking by detecting passive targets up to 30 m, using a perimeter setup with multiple cameras. However, no further details on accuracy nor the number of cameras are given to cover this volume. Furthermore, as the most cost efficient systems of the above mentioned, one Prime41 camera still costs about 5000 Euro. A minimal four-camera perimeter setup results in pure camera costs of 20,000 Euro (without software), which is a multiple of our complete system costs.

For tracking in larger, unconstrained indoor environments, such as tunnels and mines, examples of application of optical tracking systems are rare and only exist for highly special measurement purposes. One example is the application of a hand-held digital camera in combination with fixed installed visual markers for monitoring tunnel wall displacements by close-range photogrammetry [27,28]. The system requires huge installation effort and therefore is not practical for daily application. A further example is the use of a tracking camera and retro-reflecting targets to track the relative position between two shields of a double shield tunnel boring machine as part of a guidance system. The system is in use in several tunnel projects and reported to function properly [29]. However, both optical tracking systems are not designed to simultaneously track several targets over longer distances in real-time.

Summarizing, existing Outside-In optical systems rely on artificial features for model-based tracking and are thus robust against environments with non-distinctive geometric structure and poor illumination. However, for wide area tracking they require a complex system setup and thus are cost intensive. Furthermore, existing NIR tracking technology remains to be highly sensitive to ambient interfering lights and infrared radiation, especially during camera calibration, making those systems incapable of being deployed in unconstrained indoor environments.

#### 1.2.3. Laser Measurement Systems

For determining the 3D position of objects with very high accuracy, classical surveying methodology such as laser measurement systems are widely applied in research and industry. The employed instruments (total stations, terrestrial laser scanners and laser trackers) simultaneously measure the horizontal and vertical angle to the target-point together with the slope distance by using laser distance measurement. Based on these polar observations, the 3D coordinates of the target-point are then processed. Depending on the specific surveying task, the target-point is either a geodetic prism or a non-signalized point, directly located on the object surface (reflector-less measurement). The most frequently used instrument type is the total station [30,31]. In the application field, it can be found manually operated as well as integrated in automatic measurement and mobile multi-sensor systems. Advanced total stations have the capability to automatically search for, recognize, measure and even lock a prism, thus, are able to follow a slowly moving object. These options are primarily used to facilitate manual operation, increase speed of work and are indispensable when kinematic surveying is to be performed. Total stations are highly accurate for large distances of 100 m and more. They are used for setting out, network measurements, tunnel heading control, machine guidance and displacement monitoring. However, specialized personnel are required for instrument control and several (kinematic) visual objects cannot be simultaneously sighted and measured.

The technology of laser scanning by use of Terrestrial Laser Scanners (TLS) [32] is also broadly common in underground construction. It is routinely applied for a variety of purposes, such as tunnel profile control, volume determination and check of tunnel surface quality [33,34,35]. Recent research work [36] aims to use the technology for monitoring of tunnel wall displacements. As with total stations, laser scanners are operated either manually or automatically when integrated in tunnel laser scanning systems. They can perform static and kinematic scanning. However, the technology requires extensive post-processing of 3D point clouds and does not allow for efficient measurement of defined points or objects with low latency. So far, the technology does not provide real-time capability. Recently, Leica Geosystems introduced an approach that integrates an optical tracking system with a laser tracker [37,38]. It offers automatic lock-on and tracking of the 3D position (by the laser tracker) and 3D orientation (by the optical tracking system) of a hand-held target [39] with high precision and low latency up to 18 m. As a portable system, it is designed for industrial applications (e.g., prototyping and reverse engineering, tooling inspection and part mating, positioning and aligning of machines). By using a special corner cube reflector, the range can be extended up to 160 m but only for the laser tracker, not for the optical tracking system. However, up to now, this system is only used for very particular measurement tasks in tunnel construction. The only example of regular use is the check of tunnel segment geometry, a daily task performed in the segment factory. Up to now, laser trackers cannot be found underground as they are expensive and not considered robust enough to operate in harsh environments. Besides, they cannot simultaneously track multiple targets.

## 2. Methodology

Although prior art in infrared optical tracking systems lack the capabilities of robust wide-area 3D position estimation, the underlying technology is very promising to achieve our goals since it offers high precision with very low latency. Therefore, we extend this technology to overcome limitations of state of the art systems. In this section, we describe the proposed wide-area *Outside-Looking-In* optical tracking system for 3D position estimation that is based on a stereo camera setup to track targets up to distances of 30–100 m, depending on the tracking task. It provides high tracking accuracy while being robust against interfering lights during calibration and tracking.

### 2.1. System Requirements

To achieve the outlined research goals the following requirements were specified to be fulfilled by the tracking system:

- Cover Wide Tracking Volume: Target(s) shall be tracked with two cameras up to distances of 100 m. To account for different real-life tracking scenarios, the distance between both cameras (baseline) may vary. Both cameras are connected to one processing unit, thus data exchange interfaces are required that support long distance cable transmission.
- Accurate Camera Calibration: To optimally compensate optical aberrations, the intrinsic and extrinsic calibration must be able to be performed with the complete camera encasement. The extrinsic calibration has to be capable to be performed during on-going activities in the tracking volume and thus must be able to cope with heavy interferences and large distances.
- Unique Target Identification: Interfering light sources must be filtered to allow for a robust target detection during calibration and tracking, as illustrated in Figure 1b.
- Continuous and Accurate 3D Position: The hardware and software algorithms have to ensure precise target detection at large distances and in environments with poor visibility due to particles (dust, dirt) in the air. Continuous 3D position estimation must be provided within the whole tracking volume.
- Robust Hardware Casing: To ensure system reliability in real-life environments, hardware components (cameras, lenses, target, processing unit) have to be encased to be dust- and dampness proof. Nevertheless, the system must be easy and quick to setup and the target should be usable even with thick gloves. Furthermore, side effects on the camera’s field-of-view (FOV) as well as optical aberrations must be considered when encasing the vision parts of the system.

### 2.2. Algorithmic Overview

**Figure 2:**
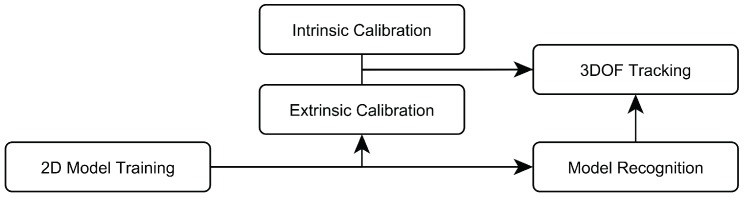
Overview over the system’s workflow.

Based on the system’s requirements, a 3D position estimation pipeline using stereo image pairs was designed. Its overall work-flow is depicted in Figure 2. In the following sections, each module and the underlying algorithmic approach are described.

### 2.3. Target Design

Within the whole intended tracking volume, the target must be reliably visible in the cameras’ images to ensure robust feature segmentation. The target’s optical features can either be arranged on a planar surface or consist of spherical optical markers so that their 2D representations in the camera image (*Blob*) are defined by circles whose centroids are computed. These spherical optical markers can either be passive—reflecting infrared light that is strobed into the tracking volume back to the camera—or active—light sources that emit towards the cameras and must be individually powered.

**Figure 3:**
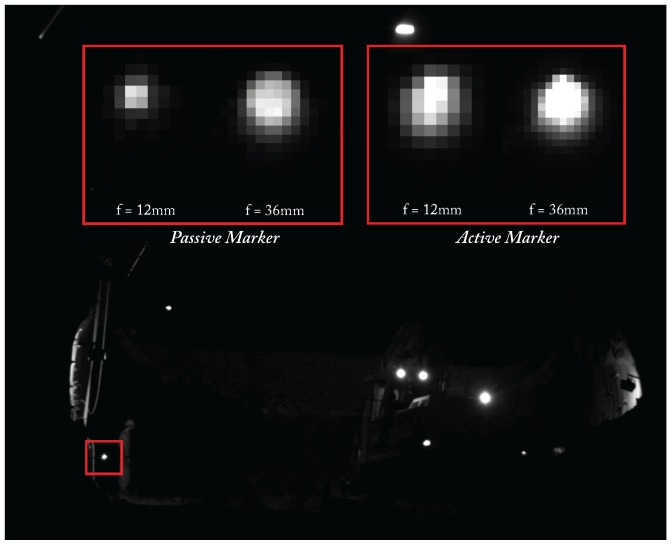
Blobs at 50 m distance with minimal/maximal focal length of f=12/36 mm.

In a preliminary study [40], we practically evaluated passive and active targets at large distances in a harsh environment (a tunnel during on-going construction). Test images have been captured using a high machine vision camera (1/1.8" Mono CCD, 1624×1224 px), an IR long-wave pass filter, a vari-focal lens (focal lengths *f* = 12,36 mm, aperture = F2.8) with 8 bit pixel depth at distances of 30 m, 50 m and 70 m, employing open aperture (f/2.8). We defined a blob to be robustly detectable if it features 80%–100% of the maximal luminance [23]. As passive targets, retro reflective foil targets in combination with a 850 nm illuminator were employed. The active target comprised an infrared light emitting diode (IR-LED) with a peak wavelength at 850 nm and a viewing half-angle of 23${}^{\circ }$. As illustrated in Figure 3, passive as well as active targets were robustly segmented in the camera image up to a distance of 50 m. At a distance of 70 m, blobs of passive targets could not be robustly detected while active targets were still visible and could be accurately segmented despite dust and dirt in the air. Consequently, active targets are suitable to fulfill the proposed system’s objectives.

**Figure 4:**
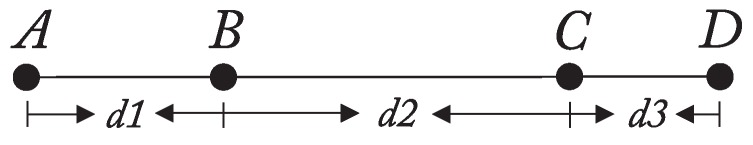
The 2D model design features projective invariant properties.

Based on these findings, we propose to use IR-LEDs as optical markers to assure precise feature segmentation in scenarios with interferences as well as at large distances. To protect the IR-LEDs and to prevent optical aberrations (flare artifacts on the blob edges in the camera images), each IR-LED is covered with a translucent diffuse plastic sphere. To detect the targets’ blobs amongst interfering lights in the camera image, the active optical markers are arranged as a line as geometric constellation, as illustrated in Figure 4. It consists of four collinear IR-LEDs which are attached in fixed distances d1,d2,d3 to each other, forming a *Rigid Body Target*.

Four points along a line comprises two projective invariant properties, cross ratio and collinearity [41]. These target model properties can be exploited for robust *Model Fitting* within the camera images, as described in Section 2.5, as well as for occlusion recovery, as explained in Section 2.7. *Model Fitting* is known as the problem of determining the correspondences between the detected 2D image features and the optical features of the tracked object. It can be accomplished by matching and fitting the detected 2D image features to the underlying target model that describes the structure of features on the tracked object. With the proposed line approach, different unique constellations can be easily designed to distinguish between multiple targets in the same tracking volume. Existing 3D rigid body targets (e.g., [42]) also offer permutation invariant geometric constellations to track multiple targets. However, enabling *Model Fitting* in 2D has three advantages over 3D approaches that are crucial for our intended research goals:

1. The line target can robustly detected across both camera images with no a priori knowledge of the camera’s epipolar geometry. Thereby, extrinsic camera calibration can be performed even in the presence of interfering lights by using the visual features of the target. Competing approaches [21,22,23] perform *Model Fitting* in 3D space, making these systems not able to cope with unconstrained conditions during camera calibration.
2. We can re-purpose the tracking target as calibration apparatus since *Model Fitting* is always performed in the 2D image domain. Thereby, the overall amount of necessary hardware for setup and maintenance can be reduced.
3. Fixing the IR-LEDs in a 2D manner increases the physical robustness of the target against accidental breaking off when touching the target during usage; this is especially an issue for tracking at larger distances since the target requires enlarged dimensions as well. Accidental breaking off is a common problem with the rather sensitive 3D rigid targets that need frequent replacement or repair by experts.

### 2.4. Camera Calibration

The proposed stereo camera rig must be calibrated to perform precise feature segmentation and to provide 3D point reconstruction of the target model’s IR-LEDs. Determining both the intrinsic and extrinsic camera parameters yield the process of *Camera Calibration*. For each camera that is involved in this stereo setup, both parameter sets are described by the *Camera Projection Matrix* *P* [17].

#### 2.4.1. Background

A number of calibration approaches exist, and all share the common principle of determining the cameras’ parameter by initially obtaining a specific number of 3D world→2D image point relations, to later use these relationships in an optimization procedure. The existing approaches for multiple view camera calibration can be categorized based on the applied calibration object and its dimensionality. Calibration based on a 2D or 3D reference target usually observes the object that is only shown at a few different orientations [43,44] undergoing an unknown translation. The object’s 2D, respective 3D geometry, is known with high precision. In 2D, this is typically a planar pattern and in 3D two or three planar pattern in an orthogonal geometric arrangement to each other. With such a reference object, each cameras’ internal (focal length, principal point offset, aspect ratio, radial and tangential distortion coefficients) and external parameters (position and orientation) can be computed efficiently [41]. Multiple view camera calibration can also be performed with a 0D object, such as corresponding points across the views. These points can be manually generated by waving a single point [45,46]—such as a light emitting diode or retro-reflective sphere—through the volume. Alternatively, these points are determined by extracting natural features [47,48,49,50] from the observed scene, which is referred to as *Auto-Calibration*. The single point methods cannot account for estimation of distortion coefficients, they only recover the extrinsic parameters. To achieve intrinsic parameter estimation, the single point methods can be combined with a 2D planar pattern calibration that is applied in advance. A sufficient number of corresponding image points (a minimum of seven is required) must be generated by a moving apertures or natural features to be able to estimate the Fundamental Matrix *F* [17,51]. The extrinsic camera parameters can then be derived—up to a scale factor—from *F* through its encapsulated epipolar geometry. To overcome the limitations of the calibration based on multiple single points, [52] presents a bar with optical markers at both ends as calibration apparatus for which the physical distance between the spheres is known. Thereby, internal and external camera parameters can be linearly determined in an initialization step, and then refined with a nonlinear least squares optimization method. Furthermore, the scale factor can be determined from the real and known distance between both spheres.

#### 2.4.2. Intrinsic Calibration

In the presented system, the intrinsic and extrinsic camera calibration is split into two steps. The intrinsic parameters, described by *Camera Calibration Matrix* *K* [17], are estimated using a planar apparatus (2D feature), a chessboard pattern. This approach is known to be robust and targets can be quickly constructed. To enhance the estimation of the parameters, all optical components (camera with lens and infrared filter) of the final tracking setup should be included in the calibration procedure.

**Figure 5:**
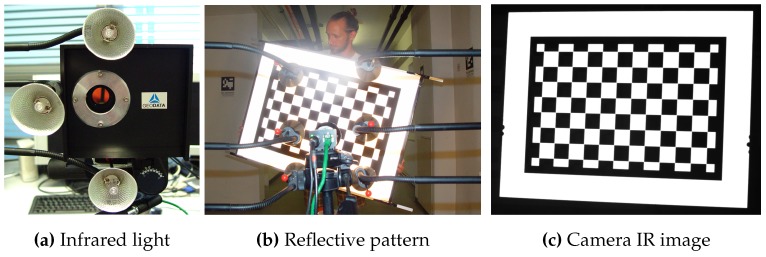
Intrinsic camera calibration with a retro-reflective pattern.

However, with such a setup, a normal black/white chessboard pattern would not be visible in the camera image. Therefore, we extended the standard intrinsic calibration setup by developing a chessboard plane made of retro-reflective foil that is illuminated with an infrared light source to provide chessboard images in the NIR (Near Infrared) spectrum. The Camera Calibration Toolbox [53] is used for intrinsic parameter determination. The complete intrinsic setup is illustrated in Figure 5. Since the lens configuration must not change after intrinsic calibration and the tracking will be at large distances, the focus settings are set to unlimited that results in a blurred pattern at close ranges. With this setting, the images of the tracking system’s cameras and lenses (see Section 3.1) are in focus from 4 m onwards. Thus, the pattern must have a sufficient size to cover the entire camera image at a distance of 4 m. Furthermore, the sharpness of calibration images was enhanced by decreasing the aperture (f/8) for increased depth of field.

#### 2.4.3. Extrinsic Calibration

Due to the intended calibration scenario with wide camera baseline, varying ambient illumination and little distinct geometric structures, we had to omit extrinsic camera calibration methods employing a 2D or 3D calibration apparatus. This would have resulted in a large target to be visible at distances of 10–70 m while being planar to provide precise corner extraction. Furthermore, its surface would have to be composed of retro-reflective foil, which is sensitive and requires additional hardware for pattern illumination. Such an apparatus would neither be transportable nor suitable. In addition, methods based on 0D natural point features are not applicable as well since they require well-distributed features throughout the entire tracking volume to function robustly. This can be easily true in cluttered and well-illuminated environments but is hard to achieve in rather dark environments or scenarios with little geometric structures.

The proposed calibration approach uses artificial points that are created by manually waving the calibration target through the volume to achieve a high amount of detectable features. To allow for calibration in unconstrained environments with interfering lights, methods using solely a single point [23,24,45,46] are not sufficient. Those approaches require to manually mask interferences in a trained background images to avoid false positive feature correspondences; obviously, those techniques cannot cope with moving interfering lights. The approach [52] tries to overcome this limitation by evaluating the screen-space coordinates of two blobs—that corresponding physical markers have a known distance—over a sequence of camera images. To find the image correspondences, the algorithms seeks for the two longest paths of possible marker motion in each camera image and assumes that no other reflections or markers are moved through the entire working volume in a similar manner as the calibration apparatus. Using the corresponding image points the Essential Matrix *E* is estimated by performing the *Nominalized 8-Point Algorithm* [17,51]. Furthermore, this methods determines the scale factor from the known distance between both optical markers. Compared to [23,46]), the affine transformation to obtain real-world distance units [mm] is not only computed once—that can result in inaccurate tracking at larger distances—but takes the measured distance between both optical markers of each processed camera frame into account.

(1) $$\begin{array}{c} scale=\frac{d_{real}}{d_{mean}} \end{array}$$

where $d_{real}$ is the real known distance between the two markers and $d_{mean}$ is the mean distance calculated based on all measured distances between the two markers over all observed image frames. When deriving the *Camera Projection Matrix P* from the epipolar geometry, the scale can then be applied by re-formulating *t* as

(2) $$\begin{array}{c} t_{metric}=t\cdot scale,\;P^{\prime }=\left[R|t_{metric}\right] \end{array}$$

**Figure 6:**
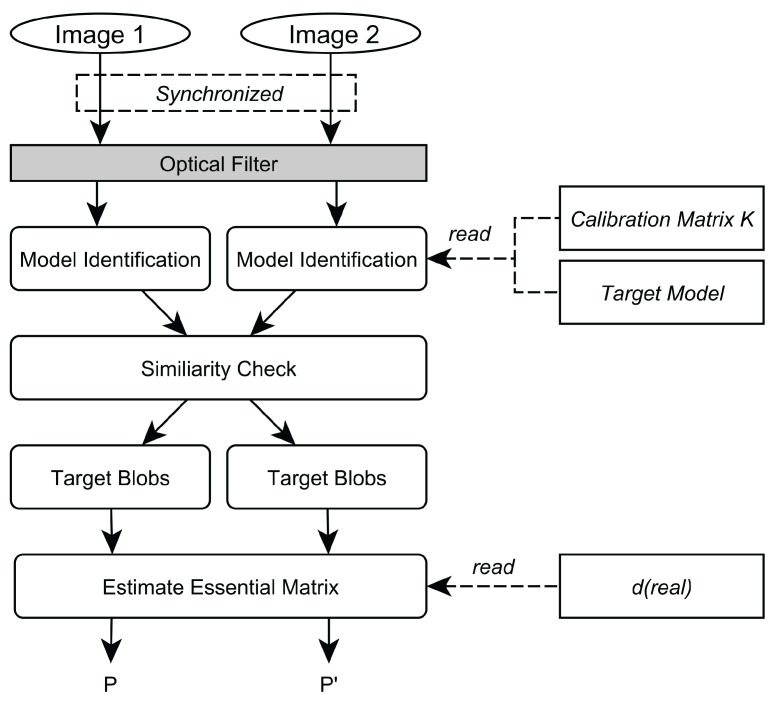
Extrinsic calibration pipeline.

However, this approach is prone to error in the presence of interfering lights in the tracking volume and in the case of short marker tracks. Thus, we extended the approach [52] and developed a pipeline—as illustrated in Figure 6—to further expunge any assumptions of marker movement and to allow single point pair correspondences.

A line target, as described in Section 2.3, is used as calibration apparatus. Its pattern can already be recognized in a 2D camera image, thus no epipolar geometry is necessary to provide correct point correspondences for the estimation of *E*. During calibration, interferences are filtered and the target is identified (*Model Identification*) using the developed pipeline from Section 2.5. This pipeline returns a set of four ordered points *p* for each camera *L* and *R* of a frame at time *t*.

(3) $$\begin{array}{c} S_{L}^{t}=\left\{p_{L,1}^{t},p_{L,2}^{t},p_{L,3}^{t},p_{L,4}^{t}\right\},\;S_{R}^{t}=\left\{p_{R,1}^{t},p_{R,2}^{t},p_{R,3}^{t},p_{R,4}^{t}\right\}\\ \text{where}\;p_{L,i}^{t}\;,\;p_{R,i}^{t}\in R^{2},\;i=1...4\; \end{array}$$

Although the model fitting is reliable, the target detection in each image is still independent from each other. Thereby, errors can occur such as a false positive identification in camera 1 and a hit in camera 2, or a hit in camera 1 and no detection in camera 2 (due to occlusions). Such erroneous input data would decrease the stability of the estimation of *E* and thus should be avoided. Therefore, a *Similarity Check* between both sets $S_{L}^{t},\;S_{R}^{t}$ is performed. Since the epipolar geometry is not known yet, stereo correspondence search along the epipolar lines can not be exploited [17]. The proposed similarity check is based on the idea, that the detected target has a similar orientation in both images at time *t* up to a threshold, depending on the camera setup. For the similarity evaluation, the target in the left image is considered as a vector ${\vec{v}}_{L}=\bar{p_{L,1},p_{L,4}}$, respectively ${\vec{v}}_{R}$ in the right image. The angles $\left(\phi_{x},\;\phi_{y}\right)$ between $\vec{v}$ and the *x*-axis, respectively the *y*-axis, are determined for the left and the right image. Outliers are detected if the angles differ by more than a given threshold *λ*, as in Equation (4). The same is done for the *y*-axis. Thereby, the algorithm can be used on images taken from both horizontally and vertically aligned cameras.

(4) $$\begin{array}{c} outlier=\left\{\begin{array}{cc} {\vec{v}}_{L},\;{\vec{v}}_{R} & \text{if}\;|\phi_{x,L}-\phi_{x,R}|>\lambda \\ 0 & \text{otherwise} \end{array}\right. \end{array}$$

If outliers have been detected, the point sets $\left(S_{L}^{t},\;S_{R}^{t}\right)$ are rejected, if not, the sets are considered as correct target blobs and are fed into the calibration routine. Since *K* is known from Section 2.4.2, the *Normalized 8-Point Algorithm* is applied for computation of the Essential Matrix *E* to enhance the stability of the epipolar geometry estimation [17]. To obtain a metric scale for Equation (2), the distance between the two outermost IR-LEDs of the calibration target are measured to sub-millimeter accuracy with a high precision total station, yielding $d_{real}$.

With our described pipeline, we achieve a robust calibration procedure that can be performed in the presence of static and moving light sources. No pre-conditioning of the volume is necessary and background training as well as manual masking can be omitted, which increases the system’s ease of use during setup and maintenance. Furthermore, by re-using a tracking target for extrinsic calibration and scale estimation, additional equipment can be minimized.

### 2.5. Interference Filtering

**Figure 7:**
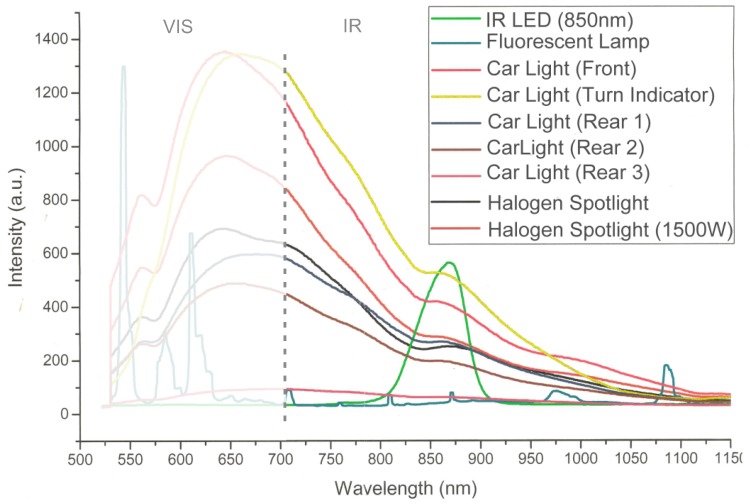
Wavelengths of various light sources.

To provide robust target identification at each stage of a optical tracking system work-flow (extrinsic calibration, target tracking), static and moving interfering lights must be robustly filtered out. In unconstrained tracking environments, a varying number of ambient light sources (wall illumination, spot lights, reflections, vehicle lights, ...) might exist. To evaluate the wavelength emission, we measured frequently occurring standard illumination sources with a spectrograph. Their emission curves are illustrated in Figure 7. As depicted, almost all ambient light sources show infrared radiation. A portion of the interferences can be filtered by inserting a longwave pass filter with a cut-on value of 780 nm into the optical path. However, most of the interfering lights are still visible in the camera images and result in bright circular blobs, similar to the IR-LEDs from the target model.

To robustly detect the target amongst static and moving ambient interfering lights, we developed a software-based identification pipeline, as described in Section 2.5.2. It is built around the 2D model fitting approach that exploits the aforementioned permutation and perspective invariant properties of the target design. The target model must be thus trained once before it can be recognized during calibration and tracking.

#### 2.5.1. Model Training

**Figure 8:**
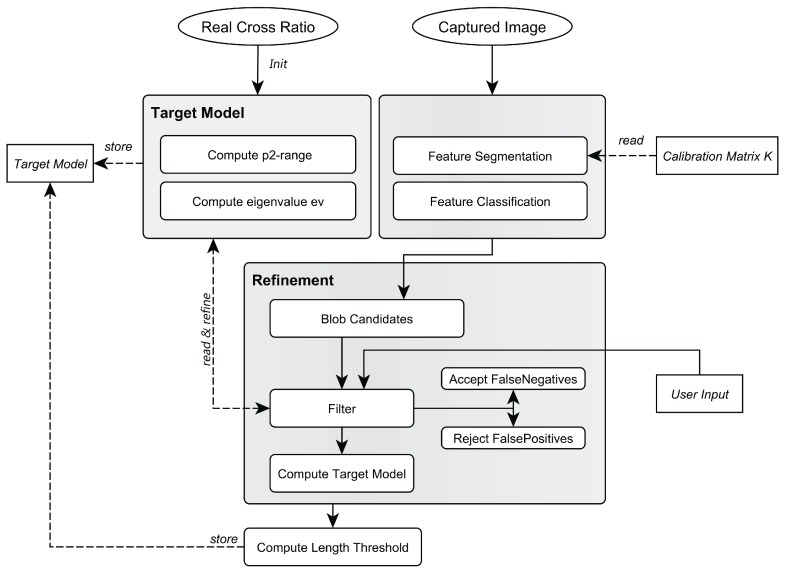
Pipeline to obtain the target’s model.

To obtain the unique properties of a target pattern, it is trained once in an off-line process to determine its *Model*, as illustrated in Figure 8.

**Figure 9:**
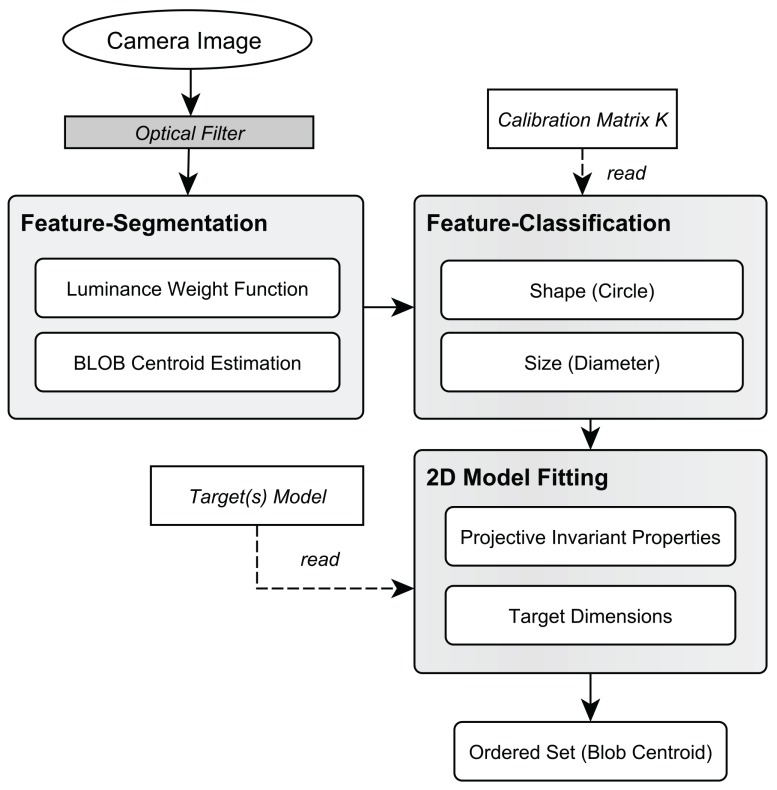
Pipeline for model identification.

The following steps—based on [54,55]—are performed:

1. The distances d1,d2,d3 (see Figure 4) between the target’s LEDs are precisely measured using a total station.
2. Based on d1,d2,d3, the cross ratio *λ* is computed and used as input argument for the function *J* to obtain an initial estimate for $p_{range}^{2}$. The eigenvalue of the moment matrix *M* as a measure for collinearity is set to an initial value such as ev≠0,ev<0.
3. The target is captured at all intended tracking distances to obtain a sufficient number of samples (images) for the complete tracking volume.
4. Each of the captured images is then processed and blob candidates are obtained by performing feature segmentation and classification (see Figure 9). $p_{range}^{2}$ and ev are applied to the blob candidates and subsequently refined to account for noise of cross ratio and collinearity.
5. After the refinement phase, the minimum and maximum length of the target in the 2D images over all images are measured to obtain a threshold $th_{range}$.
6. Finally, the obtained model is stored, containing $p_{range}^{2}$ as the minimum and maximum values of the pattern’s $p^{2}$-invariants, ev, as the collinearity error model and $th_{range}$.

#### 2.5.2. Model Identification

The pipeline of model identification is illustrated in Figure 9. After a new image (*frame*) is captured from the camera with the attached long-wave pass filter, all blobs are segmented (*Feature Segmentation*) as proposed in [23]. First, the camera image is transformed to a binary image using a dynamic threshold. Blobs are created by applying a connected component analysis as well as a circular Hough transform [56]. Next, the center of each blob (centroid) is determined using a luminance-weighted average of the connected pixels, which describe the blob’s 2D position with sub-pixel accuracy. For further processing, the centroids are undistorted based on the *Camera Calibration Matrix* *K*.

In the next step, each resulting blob is classified by performing shape- and size-based classification (*Feature Classification*). The minimum and maximum values for the size-filter can be manually defined to provide quick configuration for different tracking ranges. The classification results in circular-shaped blobs (*Blob Candidates*) that diameters lie within the specified range. In practice however further filtering must be performed since interfering lights can have a similar size as the target’s IR-LED blobs. Based on approaches [54,57,58,59], a 2D *Model Fitting* within the set of remaining blob candidates is performed. Therefore, the $p^{2}$-Invariants of the blob candidates as well as their collinear properties are computed and compared to the pre-calculated target model. Thereby, false positive blob candidates are rejected and the target’s blobs are determined. Due to the permutation invariant properties of the computed $p^{2}$-invariants, an ordered set of blobs $S^{t}=\left\{p_{i}^{t}\right\},i=1...N,p\in R^{2}$ for each image at time *t* is output to be further used for calibration or tracking.

### 2.6. 3 Degree-Of-Freedom Tracking

**Figure 10:**
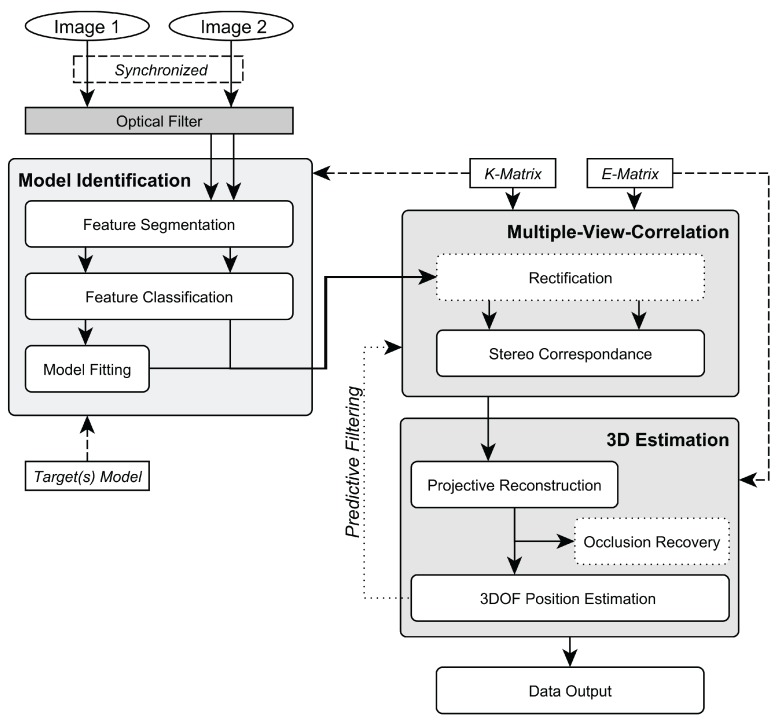
Tracking pipeline.

To track optical markers in 3D space, the following two problems have to be solved: (1) the 2D blobs have to be identified throughout all camera views and then transformed to 3D marker locations, and (2) the 3D markers need to be tracked through time. The online image-processing pipeline for tracking is depicted in Figure 10.

Given an intrinsically and extrinsically calibrated, shutter-synchronized stereo camera rig, the tracking is performed as follows. After a new frame is received from each camera, blob candidates are segmented and classified in both frames, as described above. Next, the aforementioned 2D model fitting approach is applied within the camera image to detect the target pattern. The 3D position of the pattern’s optical marker are only computed if and after the pattern was found in the image. To minimize computational load, the model identification is only performed in *Image 1* by applying model fitting within the set of all blob candidates. After the target blobs have been determined in *Image 1*, their correspondences have to be identified in *Image 2* amongst all blob candidates that result from the feature classification by exploiting the epipolar geometry, which is encapsulated in *E*. For each target blob in *Image 1*, a search for its corresponding blob is performed along its epipolar line (*Stereo Correspondence*) in *Image 2* [17].

By applying model fitting within the 2D projections of the target’s IR-LED not only a drastically reduced set of correspondence candidates and ambiguities is obtained but the combinatorial complexity of the multiple-view correlation problem can be considerably decreased as well. By performing a projective triangulation between each correlated 2D blob-tuple (*Projective Reconstruction*), the 3D-coordinate of each optical marker can be reconstructed. Following [23], we apply the standard *Singular Value Decomposition* (SVD) to obtain the initial 3D estimate for each blob-tuple, followed by bundle adjustment [60] with a Levenberg-Marquardt non-linear least squares algorithm for refinement. This results in a 3D point cloud of the reconstructed model points $T=\left\{P_{1},P_{2},P_{3},P_{4}\right\},P\in R^{3}$ that is optimized using the least square approach of [61]. To further increase the algorithm’s robustness against outliers of the model fitting, the model points *T* are validated with a threshold to account for noise against the target’s geometric constraints d1,d2,d3 (see Section 2.5.1) and volume [54]. Based on *T* and a given distance $d_{epi}$ as the real distance between the outermost IR-LED and the epicenter of the target, the target’s epicenter $C\in R^{3}$ can be calculated (*Position Estimation*) as follows.

(5) $$\begin{array}{c} C=P_{4}-\left(d_{epi}*\hat{m}\right) \end{array}$$

Therefore, we normalize the vectors $\vec{a}=\bar{P_{2}P_{1}}$, $\vec{b}=\bar{P_{3}P_{2}}$, $\vec{c}=\bar{P_{4}P_{3}}$, resulting in $\hat{a},\hat{b},\hat{c}$. By calculating the arithmetic mean of $\hat{a},\hat{b},\hat{c}$, we determine the mean direction $\hat{m}$ which is applied according to Equation (5). Thereby, an arbitrary point along the line can be determined, resulting in the 3D pose of the target. In order to enhance the robustness when tracking the target through time, the resulting target pose can be fed into a recursive filter (*Predictive Filtering*). Thereby, jitter can be reduced and the system’s intrinsic latency can be compensated. Since we currently aim for position tracking, the non-extended Kalman Filter [62,63] is therefore employed.

### 2.7. Occlusion Recovery

If a target’s IR-LED and an interfering light source lie on the same line of sight of the camera, their corresponding blobs can overlap in the images. Furthermore, parts of the target can be occluded, *i.e.*, when the target gets partly hidden behind an object in the scene. Our model fitting approach requires four optical markers. Currently, the proposed target identification pipeline can compensate one occluded marker while retaining the capability of detecting the target within the set of blob candidates. After projective reconstruction, the 3D positions of occluded markers can be reconstructed based on the target’s geometric model and the resulting 3D point cloud. The recovery of occluded IR-LEDs optimizes the accuracy of the 3D position estimate of the target’s epicenter. With this recovery functionality, loss of tracking can be reduced in cases of occlusions or over-blooming by (stronger) interfering light sources.

## 3. System Development

Based on the methodological approach, we developed a hardware- as well as software system to test our tracking system in large, unconstrained indoor environments.

### 3.1. Hardware

**Figure 11:**
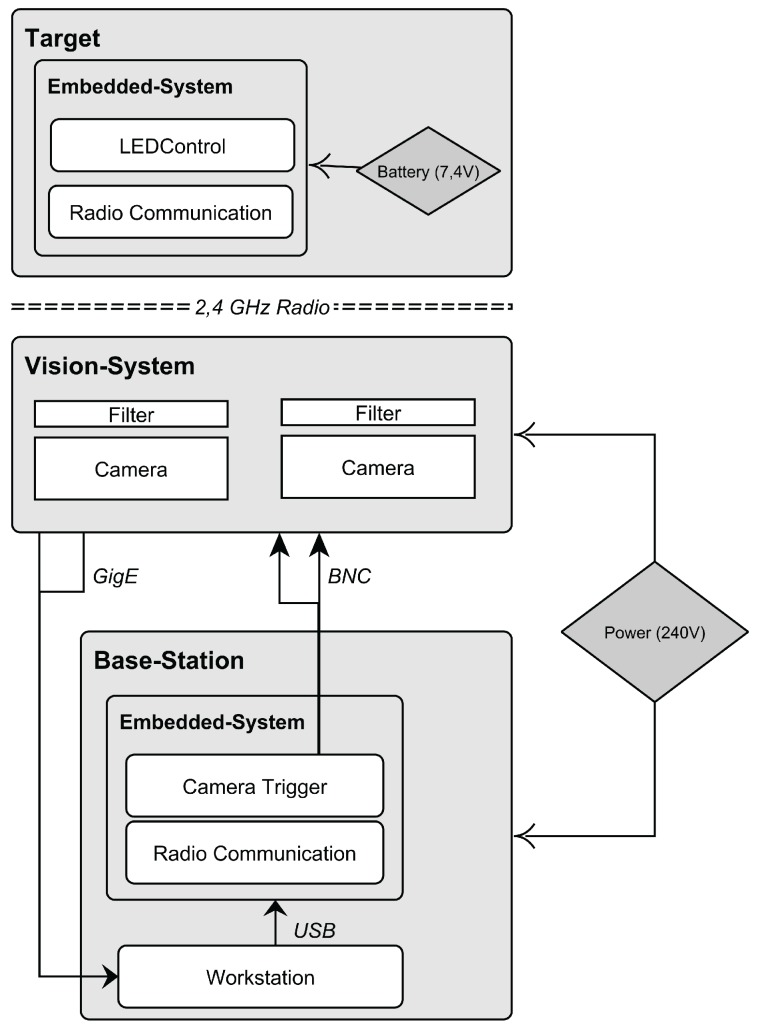
The cabling of the hardware prototype.

Our hardware prototype comprises the vision system, target(s) and a standard notebook as main processing unit. The schematics of the hardware components as well as cabling and power supply are illustrated in Figure 11.

#### 3.1.1. Vision System

The vision component of the proposed tracking system comprises two cameras, lenses and filters. To meet the system’s requirements we designed an optical setup that can cope with minimal illumination, is IR sensitive and minimizes optical aberration as well as rasterization effects while providing a sufficient field-of-view (FOV) as well as depth-of-field to cover the intended tracking volume with objects in focus. The coverage depends on focal length *f*, the distance between the cameras (baseline) as well as the amount of yaw-rotation *β* of each camera. Our system uses high-resolution machine vision cameras in combination with low-distortion lenses that feature large aperture and minimal optical aberrations. We use two Dalsa Genie HM1400/XDR cameras which feature low heat evolution and a global-shutter CMOS sensor (1” monochromatic) with high NIR spectral sensitivity. Low heat evolution and large image sensors yield little sensor noise to minimize jitter in the camera image. Together with the high resolution image sensors, precise segmentation can be provided even at longer distances. The cameras offer high global shutter speed to minimize motion blur when the target is moving fast. It is capable of delivering 60 frames per second (fps) with a resolution of 1400×1024 pixels. Both cameras are equipped with a EdmundOptics NT63-246 high-resolution and fast (f/1.4–f/16) fixed focal lens (f=25 mm). To filter light from the visible wavelength spectrum, we attached a Heliopan RG-780 long wave pass filter allowing only wavelength above 780 nm to transmit.

Both cameras form a *Stereo Camera Rig* and are shutter-synchronized by an external trigger signal to guarantee temporal synchronous image pairs. Therefore, a square-wave current loop signal is generated by the trigger unit with a built-in programmable oscillator. The trigger unit comprises two BNC (Bayonet Neill Concelman) connectors to interface with the cameras and the trigger signal, generated by an Arduino Uno board [64]. Via USB 2.0, the Arduino board connects to the mobile workstation for communication with the tracking software as well as for power supply. The Arduino Uno board furthermore interfaces with a 2.4 GHz radio module, consisting of a Nordic nRF24L01+ chip and a 5 dBi dipol-antenna. Via this radio transmission, the target’s state can be remotely controlled to provide quick system configuration during testing.

To provide wide area tracking in width and depth, the baselines can heavily vary in the intended tracking environment. Thus, data transmission from the cameras to the workstation is performed using GigE Vision [65] to guarantee lossless image transmission while providing long cable lengths. Both cameras are connected to one workstation for image processing and tracking.

#### 3.1.2. Workstation

The workstation runs the software prototype and features two Gigabyte Ethernet host adapters (1× built-in, 1× ExpressCard) to interface via ISO/IEC 11801 (Category 6) cable with the cameras. The components of the base station are centrally powered by one external 240 ACV supply.

#### 3.1.3. Target

**Figure 12:**
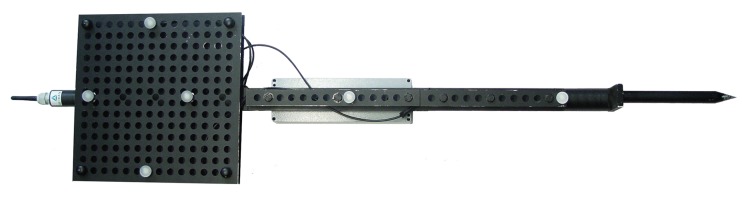
Developed target prototype.
  With the developed prototype, the 3D position of a static point can be measured. This is a common tunneling task. Since the target features a 20 cm long tip without any optical markers attached, also points that are not visible to the cameras can be tracked. Thereby, the disadvantage of vision-based tracking systems that require a line-of-sight between cameras and measured point can be compensated to a certain extent. As soon the target is freely moved in space the 3D position of the target’s tip is continuously tracked. In Figure 13, further details of the prototype are shown, including the coating of the IR-LED as well as dampness-proof cabling.

We developed three target prototypes to account for the three different evaluation scenarios: (1) User tracking for VR/AR applications; (2) Handheld target tracking for tunneling; and (3) Machine guidance for mining. Each target prototype follows the design principles from Section 2.3 and is described in the following:

- Target for Virtual Reality: For the VR/AR setup, we developed a target prototype that offers continuously adjustable positioning of the IR-LEDs by fixing each LED separately with nuts on a rigid bar to allow a rapid arrangement of the IR-LEDs. The target prototype has a total length of 687 mm and is equipped with four IR-LEDs *OSRAM 4850 E7800* in a permutation invariant constellation. Each IR-LED emits a peak wavelength of 850 nm with a radiant intensity of 40 mW/sr (mW/sr: milli watts per steradian) and features a viewing half angle of $\pm 23^{\circ }$. Thereby, robust feature segmentation up to a distance of 30 m can be performed. With the employed vision hardware setup, a minimum distance of 130 mm between two neighboring LEDs is advisable with a shutter speed of 1000 μs to avoid blob overlaps in the camera image at the maximum tracking distance of 30 m. Tracking in a smaller volume automatically leads to a decreased target size with the above mentioned setup. To further reduce the physical target size for volumes up to 30 m, LEDs with different radiant intensity properties are applicable.
  For testing, the IR-LED bar was attached to the front of a head mounted display. It has to be noted that a single line target is sufficient to determine the user’s (head) 3D position in scenarios in which the user faces the cameras, *i.e.*, in our test setup or in a semi-immersive VR scenario in which the user is tracked in front of a projector wall. In a fully immersive VR environment—where the user freely moves in space—a single line target in combination with two cameras results in occlusions as soon as the user turns around. This occlusion problem can be compensated by applying a composed setup of multiple unique line targets.
- Target for Tunneling: The target prototype was developed in cooperation with Geodata Ziviltechniker GmbH (Leoben, Austria) and is depicted in Figure 12. The target provides an array of holes at fixed distances, in which the IR-LEDs can be mounted. This allows for the rapid arrangement of multiple IR-LEDs in a permutation invariant geometric constellation. Furthermore, multiple unique constellations can be easily designed to simultaneously track one or more targets in the same tracking volume. The maximal distance between the two outermost IR-LEDs is 82.0 cm, while the targets total length is 120.0 cm. The target is equipped with six IR-LEDs *OSRAM 4850 E7800* to be able to construct a planar pattern in future as well. However, all experimental results are based on four collinear LEDs. Each IR-LED emits at a peak wavelength of 850 nm with a radiant intensity of 40 mW/sr and features a viewing half angle of $\pm 23^{\circ }$. A minimum distance of 175 mm between two neighboring LEDs with a shutter speed of 1000 μs is required to ensure robust feature segmentation up to a distance of 70 m. This distance was empirically determined with the given vision.

**Figure 13:**
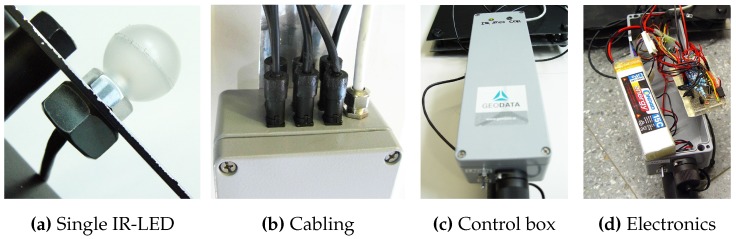
Details of the developed target prototype.
- Target for Machine Guidance: For the third evaluation scenario—machine guidance—two re-configurable targets were developed. In this scenario, a tracking range up to 100 m had to be provided. Therefore, IR-LED tests were performed to account fo the required extended tracking range. To enable reliable tracking throughout the extended tracking range, robust feature segmentation and blob centroid determination must be ensured. Therefore, different LED types from various suppliers have been evaluated at distances from 30–110 m featuring radiant intensities from 40–230 mW/sr. The aim was to find the IR-LED with the best balance between appropriate intensity for long distance feature segmentation and minimal distance between two neighboring LEDs. For all tests, the vision setup from Section 3.1.1 was employed. We ran the LEDs with $V_{F}=1.5$ V, $\;I_{F}=100$ mA and an operating voltage of 5 V. Images were captured with 8 bit, a shutter speed of 1000 μs, unlimited focus and open aperture (f/1.4). Over all tests, the IR-LED *Vishay TSHG6210* with 230 mW/sr and a half angle of $\pm 10^{\circ }$ achieved the best blob quality at large distances.
  In Figure 14, the blobs of *Vishay TSHG6210* and *OSRAM 4850 E7800* (used for the target prototypes for VR/AR user tracking and tunneling) are illustrated. The difference in luminance quality and even distribution is clearly visible. For the machine tracking prototype, a target has been constructed in cooperation with Geodata Ziviltechniker GmbH (Leoben, Austria) that consist of multiple *Vishay TSHG6210* IR-LEDs. Each LED is encased in a plastic hemisphere which acts as a light diffuser (see Figure 15b) and is installed in the center of a retro-reflecting tape target.

**Figure 14:**
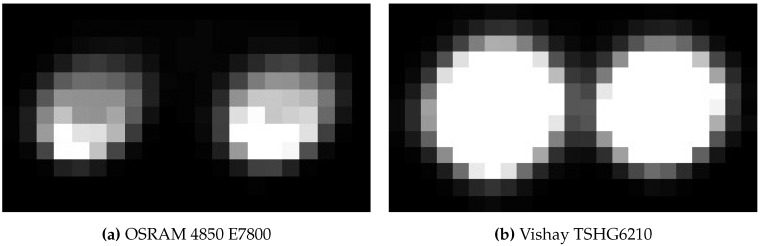
Comparison of blob quality at 110 m with an inter LED distance of 34 cm.
  The diffuser serves for an optimal light diffusion and feature segmentation as well as protects the IR-LED. The target design enables simultaneous geodetic measurement and optical tracking; thereby, the camera system’s world coordinate system can be transformed into a geodetic reference system for comparison as well as real-life use. Coordinate system transformation is subject to future work, thus this part is not covered and discussed within the proposed work. Four to five of the single IR-LEDs are combined to form a line target, as shown in Figure 15. Each single target is mounted to a 160.0 cm square bar steel and its position can be freely adjusted along the bar. The minimal LED distance is 22 cm to be able to distinguish between two neighboring LEDs at a distance of 120 m. A geodesic prism can be attached as well, as shown in Figure 15a to measure the target with a theodolite as well. We developed two of these line targets to test multiple constellations as well as simultaneous tracking. All IR-LEDs of both targets are centrally powered by one main unit, featuring battery as well as 240 Hz power supply.

**Figure 15:**
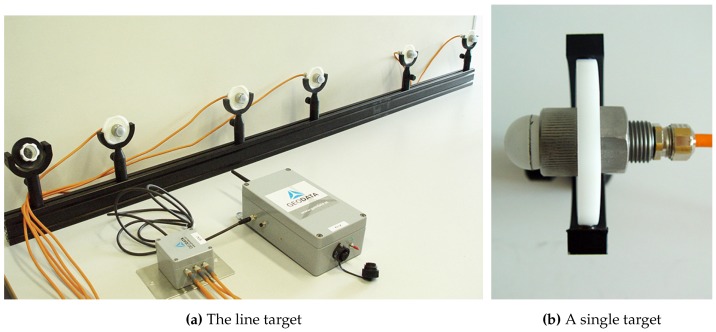
The IR-LED line target prototype for machine tracking.

#### 3.1.4. System Costs

As stated in Section 1, cost efficiency is one of the objectives of the presented tracking system. Therefore, we minimized the amount of necessary hardware and focused on off-the-shelf components as well as open source hardware and software. The current hardware prototype costs in total ∼7300 Euro, excluding camera- and target casing. The price includes both cameras (each 2000 Euro with IR filter), lenses (each 600 Euro), notebook (2000 Euro), the synchronization unit (30 Euro for Arduino, BNC adapters and cabling) and technical parts for the target (60 Euro for Arduino, radio chip, battery, wires, IR-LEDs and target material). The achieved price outperforms existing optical tracking solutions, *i.e.*, the Prime41 system [26] that yields a minimum of 20,000 Euro only for hardware to provide tracking up to 30 m.

### 3.2. Software

**Figure 16:**
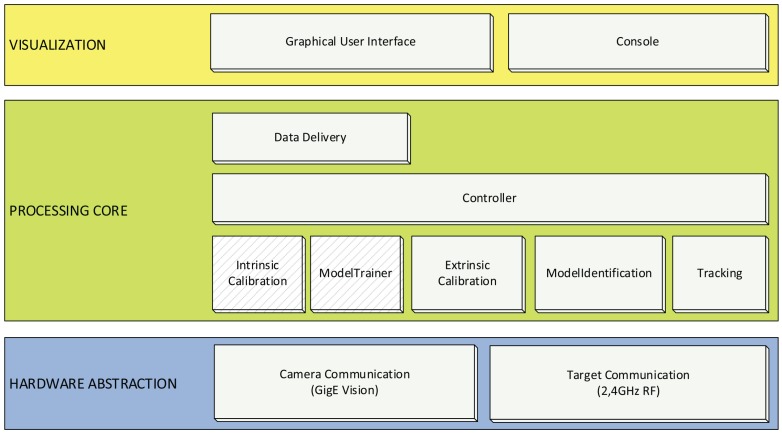
Software architecture and modules.

The developed software framework follows a three-tier-architecture comprising hardware abstraction, a processing layer and data visualization on a graphical user interface, as shown in Figure 16. The processing core consists of loosely-coupled modules for the offline processes intrinsic calibration and model training, as well as for the online processes target identification, extrinsic calibration and tracking. The modules and their functionalities are centrally accessed by the controller component that delivers data from the processing layer to the GUI. Our software framework prototype is implemented in C/C++ and MATLAB. For the intrinsic camera calibration, the open-source MATLAB Camera Calibration Toolbox [53] was integrated. With the open-source Arduino IDE [64], we developed the embedded component for camera synchronization and radio communication.

**Figure 17:**
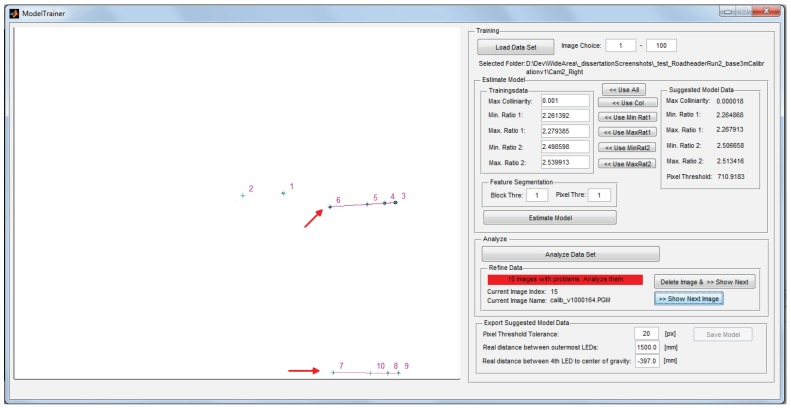
User interface of semi-autonomous Model Trainer.

**Figure 18:**
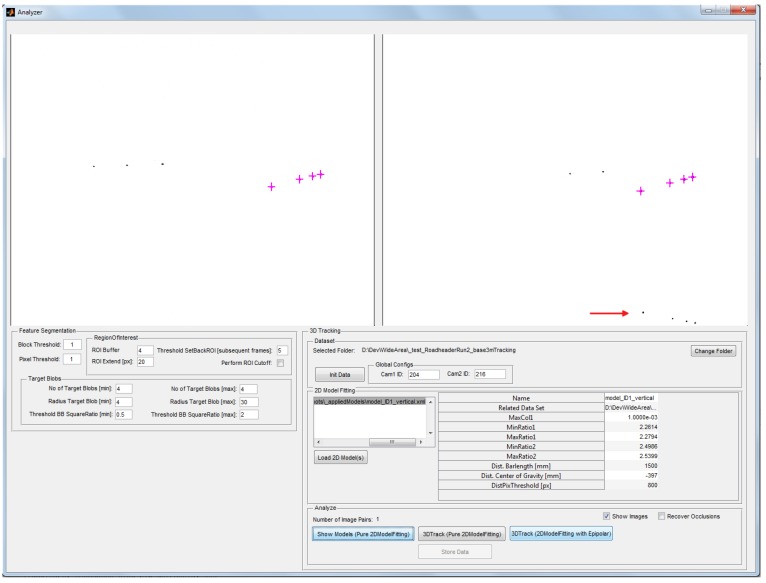
User interface of Controller to analyze data during calibration and tracking.

Training and intrinsic calibration are performed in an offline process and are implemented as stand-alone software packages. The graphical user interface of the model training component is shown in Figure 17. Please note that for visualization purpose, the camera images in Figure 17 and Figure 18 have been inverted. Based on a selected model training set, the model properties are automatically extracted and the user is informed about problems during autonomous model identification. In case of a detection of a problematic image, the user can manually adjust collinearity and $p^{2}$-invariant range or can discard the image from the training set. If no problematic training image was found, the estimated model properties are stored in a XML model file.

The image in Figure 17 shows an example of model detection in training images that have been captured in unconstrained settings. In this example, target reflections in a water puddle causes the model training to detect the target twice in the image based on the given projective invariant settings, as indicated by the red arrows. Since the model detection correctly performs with the provided collinearity and $p^{2}$-invariant range, no manual adjustment of the values is desired and the training image can be discarded from the set.

The graphical user interface of the *Controller* module for analyzing the input data during calibration and tracking is depicted in Figure 18. In this example, the same situation as in Figure 17 is shown. However, due to filtering and correspondence analysis, the blobs that are reflected in the water (indicated by the red arrow) are not considered for model fitting and subsequent tracking, demonstrating the robustness of the model identification pipeline. All parameters for hardware access, feature segmentation and model fitting and tracking are centrally stored in one XML configuration file, that can be edited and is read during system start-up.

## 4. Experimental Results

Based on the developed hard- and software prototype from Section 3, the system’s capabilities were experimentally evaluated within three different application scenarios that share the requirements of wide area tracking in an unconstrained and even harsh indoor environment:

1. User tracking for VR/AR applications
2. Handheld target tracking for tunneling
3. Machine guidance for mining

In each scenario, the robustness of target identification and the accuracy of the relative 3D position estimation was evaluated using the performance measures as described in Section 4.1. We tested our system on a Lenovo W520 notebook, featuring an Intel Quadcore i7 2820QM at 2.3 GHz, 8 GB memory and Windows7 (64 bit). The notebook acts as processing core unit that runs the software prototype. It features two Gigabyte Ethernet host adapters to interface via Category 6 cable with the cameras.

### 4.1. Test Cases and Performance Measures

The sources of error for an optical tracking system originate from a combination of optical aberrations, image processing inaccuracies as well as varying lighting situations. Since these factors potentially influence both the estimation of the external camera parameters as well as the position tracking, we separated them into two test cases in each of the three scenarios.

#### 4.1.1. Calibration Performance

Calibration performance was measured by evaluating the target identification robustness and the subsequent accuracy of the estimated relative 3D positions. Therefore, the detected blob centroids $p\in R^{2}$ in both cameras images are plotted as a function of 2D measurements over time, as defined in Equation (6).

(6) $$\begin{array}{c} f\left(x,y\right)=p_{x,y}\left(t_{k}\right),\;k=1,...,n \end{array}$$

Thereby, false positive and loss of calibration target identification, target occlusions and the feature distribution across the image are visualized and can be evaluated. The calibration performance is further examined by evaluating the relative accuracy of the estimated 3D positions. Their implicit dependency on the determined camera parameters allow for conclusions to be drawn about the quality of the calibration.

#### 4.1.2. Tracking Performance

The following measures are determined during testing to evaluate the system’s tracking performance within the three different tracking scenarios.

- Relative Position Accuracy: To obtain a valid ground truth for evaluating the relative position accuracy of the estimated 3D target position, the geometric distance between the two outermost target’s IR-LEDs is firstly measured to millimeter precision using the Leica TPS700. Thereby, ground truth $d_{bar}$ is determined. During tracking, the position of target’s IR-LEDs $L_{1}..L_{4}\in R^{3}$ are calculated for each frame *i* and used for obtaining ${\hat{d}}_{bar,i}\;=∥L_{4},L_{1}∥$, where ∥ denotes the Euclidean norm. To avoid distortion of the 3D position reconstruction, no predictive filtering is applied for testing. The estimated bar length ${\hat{d}}_{bar}$ is then applied to obtain the arithmetic mean ${\hat{\mu }}_{bar}$ with standard deviation ${\hat{\sigma }}_{bar}$ over all processed frames i=1...n, its absolute arithmetic mean deviation $|{\hat{\varepsilon }}_{bar}|$ and root mean square are denoted as follows:
  (7) $$\begin{array}{c} {\hat{d}}_{bar}\left(RMS\right)\;=\;\sqrt{\frac{1}{n}\left({\hat{d}}_{bar_{1}}^{2}+{\hat{d}}_{bar_{2}}^{2}+...+{\hat{d}}_{bar_{i}}^{2}\right)} \end{array}$$
  ${\hat{d}}_{bar}\left(RMS\right)$ is subsequently employed to obtain the deviation $x_{RMS}\left(bar\right)$, as an accuracy measure of the distance between the two outermost LEDs, and $x_{RMS}\left(P\right)$, as a measure of the relative accuracy of a single LED. Both measures are obtained as follows:
  (8) $$\begin{array}{c} x_{RMS}\left(bar\right)=d_{bar}-{\hat{d}}_{bar}\left(RMS\right)\\ x_{RMS}\left(P\right)=\frac{x_{RMS}\left(bar\right)}{2} \end{array}$$
  Thereby, the relative 3D position accuracy of a single target point can be evaluated against a ground truth throughout the tracking volume.
- Position Stability: Based on the estimated target’s IR-LED $L_{1}..L_{4}$, the target’s epicenter $C\;=\;C_{x,y,z}\in R^{3}$ is determined during tracking, as described in Section 2.6. To evaluate static jitter of the system and thus the stability (inner accuracy) of the 3D point estimation, the standard deviation $\hat{\sigma }$ of $C_{x},\;C_{y},C_{z}$ as well as *C* over the sequence of consecutive frames is calculated and used to evaluate the system’s intrinsic tracking performance.
- Tracking Latency: To obtain a measure for time-dependent tracking performance, the systems latency is measured as the time delay between the change in tracker pose and the time, the system has estimated and outputs the new tracker pose.

### 4.2. Tracking for Virtual and Augmented Reality

**Figure 19:**
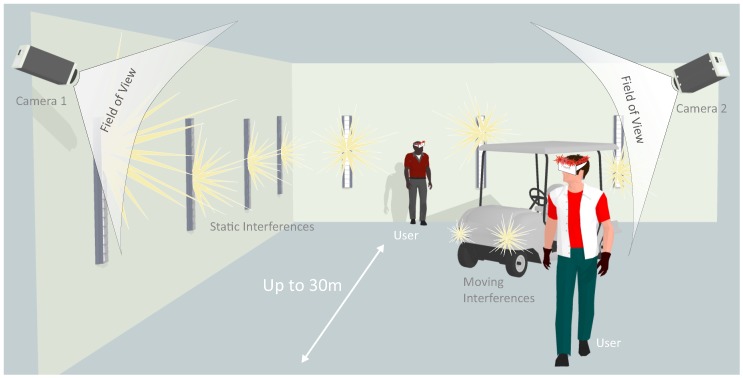
Wide area user tracking in a virtual reality setup.

Wide area user tracking can be applied to a number of application scenarios, such as user tracking in VR in environments using redirected walking approaches [66], tracking of artists on stages or personnel in workshops and factories. In Figure 19, an example scenario for user tracking in a virtual environment is depicted, that is characterized by static and moving light sources and distances up to 30 m.

#### 4.2.1. Test Environment

Since we were lacking access to an indoor environment that features the intended tracking ranges, we deployed the prototype in an outdoor environment during twilight and night. We added light sources (neon lights, halogen spots up to 1500 W) to simulate wall illuminations, reflections and locomotive interfering lights. Thereby, we established a controllable realistic simulation of the intended tracking scenario. Both calibration and tracking were performed in an environment with static as well as moving interfering lights. We employed a baseline $d_{base}\approx 10$ m and tracking distances between the vision system and target $d_{track}$ of 7.5–30.0 m.

#### 4.2.2. Model Training

As the developed target’s prototype from Section 3.1.3 is used for calibration and tracking, its model was obtained in an offline process. First, the real distances d1,d2,d3 between the target’s LEDs were precisely measured with millimeter precision using a Total Station (Leica TPS700). Afterwards, the target’s projective invariant properties were calculated by evaluating 110 captured camera images across the entire tracking volume from 5to30 m.

#### 4.2.3. Camera Calibration

**Figure 20:**
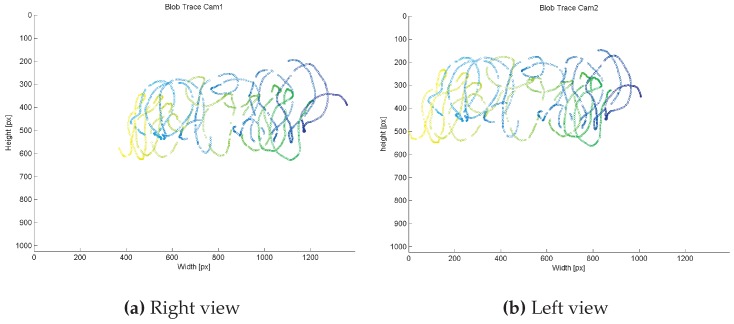
Corresponding blob traces used for extrinsic calibration.

Before setup, both cameras were intrinsically calibrated in an offline process using 34 images that captured the retro-reflective chessboard pattern from different angles and distances. For extrinsic calibration and subsequent tracking, the stereo camera system was setup with the following parameters to account for tracking distance and poor lighting situation: real baseline $d_{base}\approx 10$ m, yaw-rotation $\beta_{cam1}=30^{\circ },\;\beta_{cam2}=-30^{\circ }$, lens focus = *∞*, aperture 1.4/f, shutter speed 1000 μs. Using the proposed tracking target prototype, we performed the calibration at a distance around 15.0 m from the cameras. We ran three different calibration tests with ∼1200 frames each to evaluate the robustness of the calibration procedure. As depicted in Figure 20, our system robustly identifies the target despite static and locomotive interfering lights, resulting in continuous blob traces of the two outermost IR-LEDs. As illustrated, the blob trace was interrupted at some points due to complete occlusion of the target because of obstacles in the environment. Despite the unconstrained test calibration environment, our system robustly estimated the *Essential Matrix* *E* at each run. In average, *E* was determined with a duration of ∼110 s. The second factor for evaluating the calibration is the quality of the reconstructed 3D points. We found the calibration yielding consistent 3D point estimates for all tracking distances, as presented in detail in the following.

#### 4.2.4. 3D Position Accuracy

To evaluate the accuracy of relative 3D position estimation, we performed measurements at six different distances between camera and target, denoted as $d_{track}$ for each calibration procedure.

**Figure 21:**
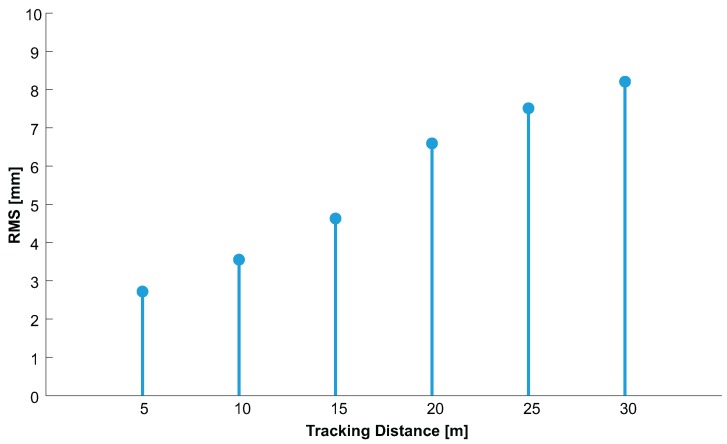
Mean of relative accuracy $x_{RMS}\left(P\right)$ over all three calibrations.

At each accuracy run, the 3D coordinate of each target’s IR-LED $L_{1}..L_{4}$ as well as of the target’s epicenter $C=C_{x,y,z}$ was estimated based on 300 consecutive frames. Thereby, accuracy and stability were evaluated for the entire tracking volume. The obtained $x_{RMS}\left(P\right)$ values for each calibration run and each tracking distance $d_{track}$ are listed in detail in Table 1. In Figure 21, the arithmetic mean of $x_{RMS}\left(P\right)$ over all three calibration runs with respect to the tracking distance is depicted.

**Table 1:** Relative accuracy $x_{RMS}\left(P\right)$ of three independent calibrations.

$d_{\mathrm{track}}$(m)	Calibration 1	Calibration 2	Calibration 3
	$x_{\mathrm{RMS}}\left(P\right)$ (mm)	$x_{\mathrm{RMS}}\left(P\right)$ (mm)	$x_{\mathrm{RMS}}\left(P\right)$ (mm)
5	3.39	2.99	1.78
10	4.12	3.91	2.63
15	4.76	4.54	4.58
20	6.08	6.23	7.47
25	6.64	6.97	8.92
30	7.44	7.96	9.22

#### 4.2.5. 3D Position Stability

To evaluate static jitter of the system and thus the stability (inner accuracy) of the system, we fixated the target and tracked it over a sequence of 200 consecutive frames. In each frame, $C_{xyz}$ was calculated to determine the empirical standard deviation ${\hat{\sigma }}_{x}$, ${\hat{\sigma }}_{y}$ and ${\hat{\sigma }}_{z}$ of the target’s center of gravity. Throughout the entire tracking volume and across the three calibration runs, we found sub-millimeter deviation for 3D position estimation with ${\hat{\sigma }}_{x}=0.05$ mm, ${\hat{\sigma }}_{y}=0.03$ mm, ${\hat{\sigma }}_{z}=0.11$ mm, resulting in an overall mean standard deviation of $\hat{\sigma }=0.06$ mm for *C*.

#### 4.2.6. Tracking Performance

**Figure 22:**
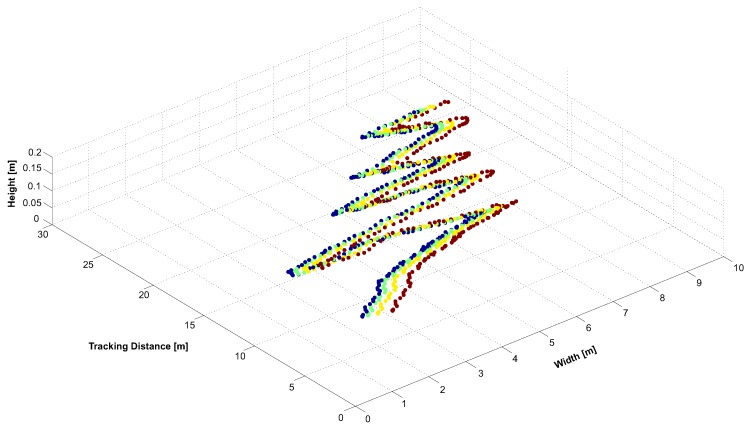
3D position tracking from 7.5–27 m.

To determine the system’s capability to continuously track a target throughout the tracking space, we moved it through the volume. The resulting 3D position reconstruction of each target’s IR-LED is illustrated in Figure 22. Depending on the number of interfering lights, our system identifies and tracks a target with a latency of ~69 ms within the unconstrained test environment.

### 4.3. Hand-held Target Tracking for Tunneling

**Figure 23:**
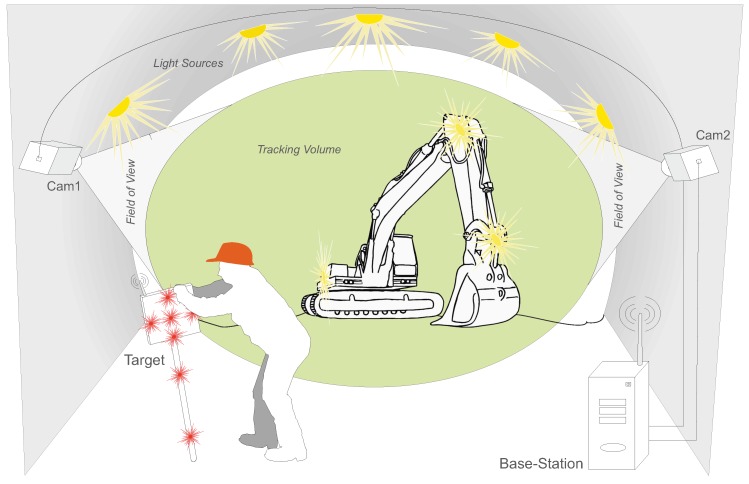
Tracking situation in an underground environment.

To further exploit the capabilities of the developed tracking system beyond application scenarios for VR/AR, it was tested in an underground scenario, using a hand-held target to track the 3D position of arbitrary static points or the moving target over time. As described in Section 1.2.3, existing technology lacks the ability of tracking a fast moving target, tracking of multiple targets as well as tracking without manual sighting. The intended underground tracking scenario, such as a tunnel or a mine, is illustrated in Figure 23. Two cameras are directed towards the tracking volume and connected to one processing unit. As soon as the hand-held target comes into sight of the cameras, tracking of the target’s 3D position automatically starts.

Compared to the previous scenario from Section 4.2, the tracking system does not only need to be able to cope with static and moving interferences, such as wall illumination and (strong) vehicles lights, but also with larger distances and harsh environmental conditions, such as dust or dirt. Dust, as a large number of small particles in the air, can influence the visibility of the target, especially at long distances, and hence decrease the quality of feature segmentation during calibration and tracking. To account for these additional challenges, a specialized hand-held target was developed and all vision components were carefully encased to enable tracking from 30–70 m.

#### 4.3.1. System Encasement

**Figure 24:**
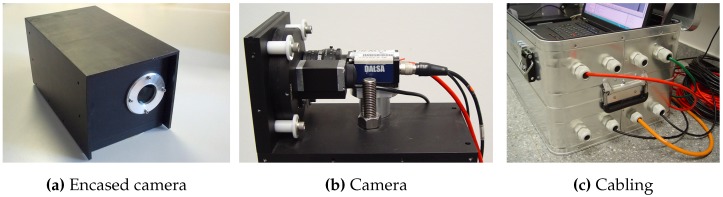
Robust and dampness proof encasement of cameras and base station.

To protect the system’s electronic parts against dampness and dust in the harsh test environment, special encasement was developed in cooperation with Geodata Ziviltechniker GmbH (Leoben, Austria). All electronic components for LED control, radio and power supply are robustly encased in the control box that features feedback LEDs to inform the user about the current tracking state. Furthermore, each camera was encased separately. The components of the base station, comprising notebook with power supply, camera trigger and radio were encased as well for protection and to be transportable and are shown in Figure 24.

#### 4.3.2. Test Environment

**Figure 25:**
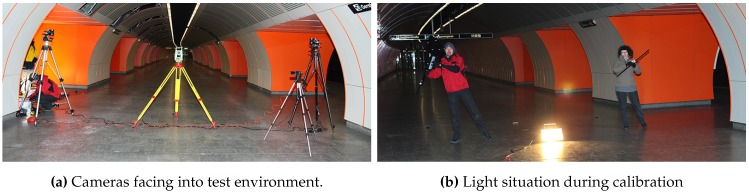
Test environment in a metro underground station.

We deployed the prototype in an underground metro station that offers a long-range tracking volume with static illumination characteristics, similar to a tunnel construction site. Furthermore, we dynamically applied moving light sources by hand, *i.e.*, halogen light up to an intensity of 1500 W, to establish a controllable and realistic simulation of the application scenario, as shown in Figure 25. Again, both calibration and tracking was performed in an environment with static as well as moving interfering lights. With respect to underground measurement scenarios, we performed calibration and tracking tests with baselines $d_{base}$ from 6–12 m and distances between the vision system and target $d_{track}$ from 30–70 m. Therefore, we prepared our test volume by measuring and marking fixed spatial points on the ground within the tracking volume in distances of $d_{track}=30,40,50,60,70$ m, using a Leica TPS700.

#### 4.3.3. Model Training

As the target’s prototype from Section 3.1.3 is used for calibration and tracking, its model again was obtained in an offline process. First, the real distances d1,d2,d3 between the target’s LEDs were precisely measured with millimeter precision using a Total Station (Leica TPS700) and $d_{bar}=820$ mm was obtained. Afterwards, the target’s properties were calculated by evaluating 205 captured camera images across the entire tracking volume from 30–70 m. To enhance robustness of the obtained model, we rotated the model during training as well.

#### 4.3.4. Camera Calibration

Before setup, both cameras were intrinsically calibrated in an offline process using 44 images captured from different angles and distances. For extrinsic calibration and subsequent tracking, the stereo camera system was setup with the following parameters to account for tracking distance and poor lighting situation: real baselines $d_{base}\approx 6$–12 m, lens focus *∞*, aperture 1.4/f, shutter speed 1000 μs.

**Figure 26:**
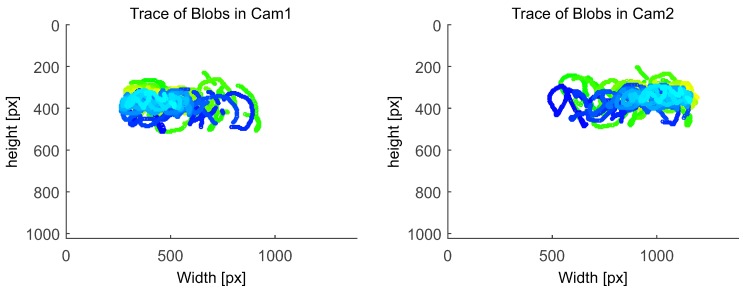
Calibration with $d_{base}\approx 6$ m.

Upon each physical re-configuration of the camera stereo system, we performed extrinsic calibrations in various distances $d_{calib}$ between camera and target—ranging from 30–70 m—with a total number of ∼1400 frames at each run. Again, our system had to continuously identify the target despite the interfering lights in the tracking volume. Figure 26 depicts the continuous feature segmentation and resulting blob traces of the two outermost IR-LEDs for a baseline $d_{base}\approx 6$ m while performing the calibration between 30–70 m.

As shown, our system robustly detects the target and can provide continuous blob traces for both cameras. For our tests at distances of 30,50,70 m the coverage of blob traces in the camera images decreases as distance between cameras and target increases. With decreasing blob coverage, a decrease in accuracy of the estimated extrinsic parameters could be observed. The calibration tests indicate the importance of well distributed blob coverage on the image to obtain an accurate extrinsic calibration result.

#### 4.3.5. Accuracy and Stability of 3D Position Estimation

**Figure 27:**
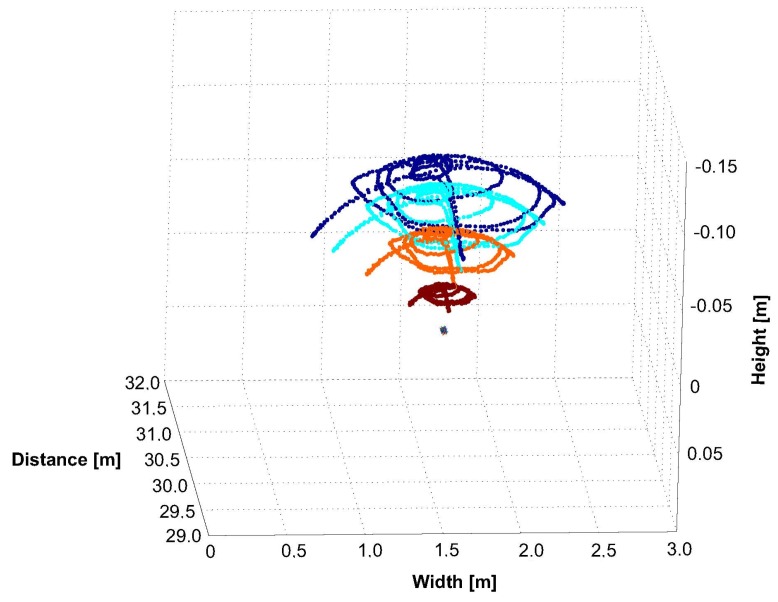
Target movement during accuracy and stability measurements.

To evaluate the accuracy and stability of the relative 3D position estimation, we fixated the target’s tip to the previously measured spatial markers on the ground. Next, we performed yaw(α), pitch(β) and roll(γ) rotations around the fixated tip over a sequence of consecutive frames. Applying these movements, we received data for an extensive and robust evaluation of the entire tracking pipeline. In Figure 27, the reconstructed 3D positions off all IR-LEDs $L_{1}..L_{4}$ and the target’s tip (epi center) *C* are visualized.

We performed six runs in varying distances, $d_{track}=30$–70 m, with two different baselines, $d_{base6}\;=\;6$ m (distance approximation = 5.95 m), $d_{base12}=12$ m (distance approximation = 12.29 m) and $d_{calib}=30$ m. Each test was running 300 consecutive frames with α,β,γ ranging from $0^{\circ }$–$45^{\circ }$. For each run, the 3D coordinates $L_{1}..L_{4}$ as well as *C* were estimated to be able to evaluate relative position accuracy by analyzing ${\hat{\mu }}_{bar}$, ${\hat{\sigma }}_{bar}$ and $|{\hat{\varepsilon }}_{bar}|$, and the stability (inner accuracy) of the 3D point, using $\hat{\sigma }\left(C\right)$.

**Figure 28:**
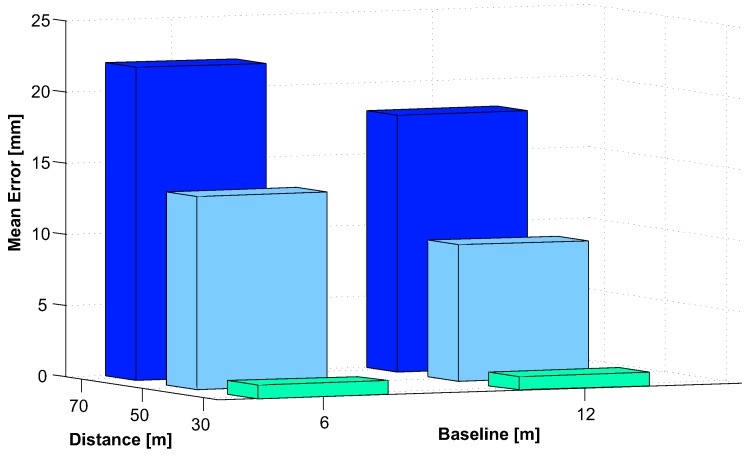
$|{\hat{\varepsilon }}_{bar}|$ for all $d_{base}$ and $d_{track}$.
  Up to 30 m with $d_{base}\approx 6$–12 m, the system is able to provide relative 3D accuracy with sub-millimeter deviation of 0.95 mm for $d_{base6}$, and 0.94 mm for $d_{base12}$. At 70 m, the system achieves 3D accuracy with a maximal deviation of 21.98 mm for $d_{base6}$, and 18.06 mm for $d_{base12}$. Hence, accuracy decreases as distance increases, and larger baselines results in better accuracy, especially at large distances. However, our evaluation for $d_{base6}$ also reveals 3D position outliers in the result set for 30 m and 50 m since as a consequence, ${\hat{\sigma }}_{bar}$ is larger. This does not indicate an overall lack of 3D position robustness since ${\hat{\sigma }}_{bar}$ is low at 30 m with $d_{base6}$ and at all distances with $d_{base12}$. Since no filtering was applied to avoid distortion of the 3D position estimation results, such outliers and its influence can be minimized application tracking using predictive filtering.
  Overall, our proposed system provides a relative 3D measurement accuracy with an absolute maximal error $|{\hat{\varepsilon }}_{bar}|=21.98$ mm (${\hat{\sigma }}_{bar}=11.04$ mm) for baselines $d_{base}\approx 6$–12 m throughout the entire volume. This accuracy has been achieved under constant movement and changes in rotation of α,β,γ up to $45^{\circ }$.
- Stability: After evaluating the accuracy of the relative position estimation, we evaluated the stability of the relative position estimation over 300 consecutive frames. Again, we continuously rotated the target by $\alpha ,\beta ,\gamma =0^{\circ }$–$45^{\circ }$. The results are shown in detail in Table 3 with respect to $d_{base}$ and $d_{track}$.

- Relative Position Accuracy: To evaluate the accuracy of the relative 3D position estimation, we performed measurements at three different distances between camera and target, denoted as $d_{track}$ for each baseline. At each run, the 3D coordinate of each target’s IR-LED $L_{1}..L_{4}$ as well as of the target’s epicenter $C=C_{x,y,z}$ were estimated based on 300 consecutive frames.
  $\varepsilon_{bar}$ with respect to both baselines $d_{base}$ and all tracking distances $d_{track}$ is depicted in Figure 28. As it can be seen for both baselines, $|{\hat{\varepsilon }}_{bar}|$ increases as $d_{track}$ increases. This is due to a more inaccurate feature segmentation at larger distances since blob size and luminance diminish. This causes bigger rasterization artifacts than in close range that reduces the accuracy of blob centroid computation. Furthermore, the distances between the blobs in the camera images decrease, especially when large rotations of $\alpha ,\beta =45^{\circ }$ are applied. With $d_{base12}$, more accurate results at larger distances can be achieved compared to $d_{base6}$. 3D point reconstruction [67,68] can be more robustly performed as the baseline $d_{base}$ increases since the glancing intersection between both rays decreases. All results of $\hat{\mu },\hat{\sigma }$ and $x_{RMS}\left(bar\right)$ are listed in detail in Table 2.

**Table 2:** Deviations and error of $d_{bar}$.

$d_{\mathrm{track}}$ (m)	$d_{\mathrm{base}}\approx 6$ m		$d_{\mathrm{base}}\approx 12$ m	
	$|{\hat{\varepsilon }}_{\mathrm{bar}}|$ (mm)	${\hat{\sigma }}_{\mathrm{bar}}$ (mm)	$\left|{\hat{\varepsilon }}_{\mathrm{bar}}\right|$ (mm)	${\hat{\sigma }}_{\mathrm{bar}}$ (mm)
30	0.95	5.29	0.94	1.54
50	13.58	14.24	9.56	3.46
70	21.98	11.04	18.06	10.09

**Table 3:** Standard deviations $\hat{\sigma }\;\left(C\right)$ at different tracking distances $d_{track}$.

$d_{\mathrm{track}}$ (m)	$d_{\mathrm{base}}\approx 6$ m			$d_{\mathrm{base}}\approx 12$ m		
	${\hat{\sigma }}_{x}$ (mm)	${\hat{\sigma }}_{y}$ (mm)	${\hat{\sigma }}_{z}$ (mm)	${\hat{\sigma }}_{x}$ (mm)	${\hat{\sigma }}_{y}$ (mm)	${\hat{\sigma }}_{z}$ (mm)
30	4.07	3.61	12.80	4.92	4.04	5.57
50	4.62	4,49	24.32	6.09	3.09	11.94
70	4.18	6.98	44.92	7.50	5.29	29.61
  The deviation of *C* correlates with the results and findings of Section 4.3.5. Above all, ${\hat{\sigma }}_{z}$ increases most as $d_{track}$ increases while ${\hat{\sigma }}_{x},{\hat{\sigma }}_{y}$ remain rather constant and are ≤7.5 mm for the entire tracking volume. Thus, tracking of the head’s 3D position is very stable for the x/y-axes with both baselines $d_{base6}$, $d_{base12}$. Our optical setup as well as the software processing results in millimeter deviation for $C_{x,y}$ with both baselines up to 70 m. These results can be improved by using image sensors with higher resolution. ${\hat{\sigma }}_{z}$ varies most at 70 m with $d_{base6}$ with a maximal deviation of 44.92 mm. With larger baselines, the 3D position estimation of $C_{z}$ gets more stable (${\hat{\sigma }}_{z}$ is decreasing for $d_{base}\approx 12$ m).

#### 4.3.6. Tracking Performance

**Figure 29:**
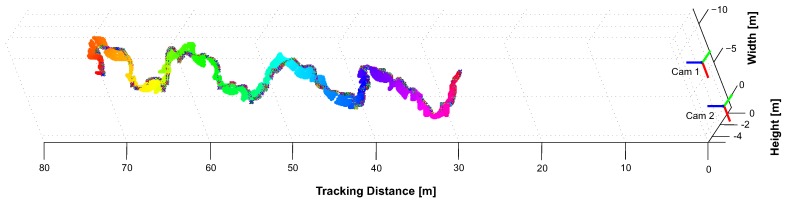
3D position tracking of a moving target through the entire volume.

Besides the accuracy and stability evaluation, we performed tests to determine the system’s capability to continuously track the target in the intended tracking space. Therefore, we moved and rotated the target through the whole volume for $d_{track}=30$–70 m and inserted static and moving interfering lights into the tracking volume. Currently the system provides ten 3D position estimates per second (10 fps). Those rates allow for interactive tracking of static and moving objects. Figure 29 illustrates the target tracking and depicts the 3D position of $L_{1}..L_{4}$ as well as *C*, indicated as cross. As illustrated, the target is robustly and continuously tracked with various rotations trough th entire tracking volume.

### 4.4. Machine Tracking for Underground Guidance

Besides the ability to measure static or moving points using a handheld target, there is a huge demand in underground construction to track machines to enable remote control. Machines such as roadheaders, jumbos, dredgers contribute to significant cost reductions and the increase of safety and efficiency of underground works.

For an efficient control of these machines the continuous and precise determination of their 3D position and orientation in the underground space is mandatory. The productivity of such machines depends on their efficient control. Therefore, an on-board machine control system is required to be able to measure, process and provide quickly, accurately and reliably all data that is needed for an optimal machine operation. One important subsystem of any such control system is the machine guidance system (navigation system) that is responsible for the determination of the absolute 3D position and orientation of a given machine and (more importantly) its different tools (e.g., booms, cutting heads) in the underground space.

#### 4.4.1. Shortcoming of Existing Technology

As described in Section 1.2.3, classical surveying methodology such as laser measurement systems are widely applied to determine the 3D position of objects with very high accuracy. Existing automatic systems use conventional tunnel lasers in combination with active laser targets/laser receivers that are installed on the machine (e.g., for jumbos). Other approaches apply classical surveying methods such as tachymetry where computer-controlled, robotic totalstations automatically and periodically measure to shutter prisms mounted on the machine (e.g., as used for roadheaders). However, the existing technologies suffer from the following shortcomings:

- They are highly specialized and designed for particular types of machines only; therefore, they lack the universal application to other machine types.
- They can only measure and thus control one machine at a time and lack the capability of tracking multiple machines as well as machine parts that simultaneously operate.
- They can only be used for the purpose of machine guidance but not also for other measuring and surveying tasks such as setting out, profile control or deformation monitoring.
- They lack real-time tracking capability, especially when using totalstations.
- They are expensive, in particular their sensor hardware.

#### 4.4.2. Test Environment

As a first approach to overcome the shortcomings of existing underground machine guidance systems, the developed tracking system from Section 2 was tested by tracking two line targets that are rigidly attached to a wheel loader.

**Figure 30:**
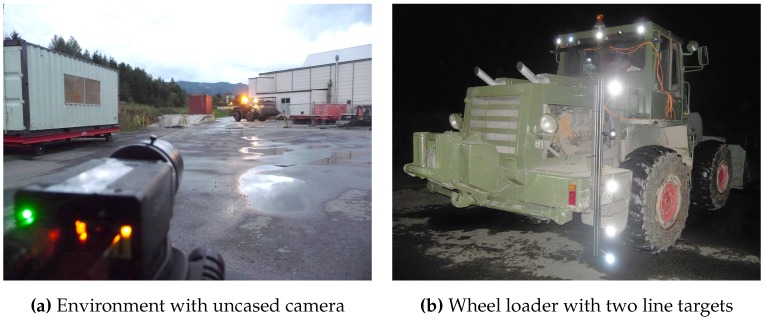
Details of the test environment.

The tests were conducted in cooperation with Geodata Ziviltechniker GmbH (Leoben, Austria) and Sandvik Mining and Construction GmbH (Zeltweg, Austria). For testing, the loader was tracked open air at twilight and night during standstill and in motion, as well as under the influence of moving interfering lights as well as artificial smoke. The described test environment is illustrated in Figure 30, the images are manually brightened by 20% for enhanced visualization. In this environment, the tracking system has to cope with additional challenges compared to Section 4.3, such as heavy vibrations of the wheel loader during movement and standstill with engine at rest, as well as with an increased tracking volume ranging from 20–110 m. With respect to underground measurement scenarios in tunnels and mines, we performed calibration and tracking tests with baselines $d_{base}$ from 3–9 m and distances between the vision system and target $d_{track}$ from 20–110 m.

#### 4.4.3. Model Training

As the target’s prototype from Section 3.1.3 is used for calibration and tracking, its model again was obtained in an offline process, as described in Section 2.5.1. First, the single LEDs of each target were set to a unique geometric constellation; then the real distances d1,d2,d3 between the target’s LEDs were measured using a total station (Leica TPS700), resulting in the following distances for *Target 1*: d1=25.0 cm, d2=40.0 cm, d3=85.0 cm; and d1=25.0 cm, d2=55.0 cm, d3=70.0 cm for *Target 2*. Hence, for both targets, the distance between the two outermost IR-LEDs $d_{bar}=150.0$ cm. Afterwards, the properties for each target were calculated by evaluating 255 captures camera images across the entire tracking volume from 20–110 m. This results in the following $p^{2}$-Invariant ranges, defined by $\left[\begin{array}{c} J_{i}^{min},J_{i}^{max} \end{array}\right]$:

$$p_{range}^{2}(Target1)=\left[\begin{array}{c} 2.2270,\;2.5200 \end{array}\right]$$

$$p_{range}^{2}(Target2)=\left[\begin{array}{c} 2.1108,\;2.1696 \end{array}\right]$$

As it can be seen, the chosen geometric constellation of both targets results in different, non-overlapping $p^{2}$-Invariant ranges. This is important for robust model identification.

#### 4.4.4. Camera Calibration

Before setup, both cameras were intrinsically calibrated in an offline process using 42 images captured from different angles and distances. The stereo camera system was setup with the following parameters to account for constrained baselines of a later application environment, the intended tracking distance as well as poor lighting situation, real baselines $d_{base}\approx 3$–9 m, lens focus *∞*, aperture 1.4/f, shutter speed 1000 μs. At each run, the system was calibrated with ∼1100 images.

#### 4.4.5. Accuracy and Stability of 3D Position Estimation

To evaluate the accuracy and the stability of the relative 3D position estimation, we performed measurements at different distances $d_{track}$ of both targets during standstill of the wheel loader with engines shut off. At each accuracy run, the 3D coordinate of each target’s IR-LED $L_{1}..L_{4}$ as well as of the target’s epicenter $C=C_{x,y,z}$ was estimated based on 180 consecutive frames at 10 fps. Thereby, accuracy and stability were evaluated for the entire tracking volume. The obtained $x_{RMS}\left(P\right)$ values as well as the empirical standard deviations of σ(C) of the horizontal target with a baseline $d_{base}\approx 9$ m are listed in Table 4.

**Table 4:** Relative point accuracy and standard deviation ${\hat{\sigma }}_{C}$ for $d_{base}\approx 9$ m.

$d_{\mathrm{track}}$ (m)	$x_{\mathrm{RMS}}\left(P\right)$ (mm)	${\hat{\sigma }}_{x}$ (mm)	${\hat{\sigma }}_{y}$ (mm)	${\hat{\sigma }}_{z}$ (mm)
20	7.28	0.19	0.12	0.73
30	17.19	0.18	0.09	0.59
40	29.04	1.57	1.28	5.86
50	42.62	0.94	0.27	3.51
60	49.04	0.56	0.23	3.16
70	49.60	0.65	0.37	4.33
80	60.72	1.05	0.59	4.71
90	78.88	0.90	0.50	5.91
100	89.31	3.70	1.28	23.53

The results of the relative point accuracy show deviations in the low cm-range throughout the volume, and up to 80 m a very high distance-invariant stability (a good repeatability of measurement results) in the low mm-range as well as even below 1 mm in the X/Y-plane (vertical cross section). As to be expected and explicable by theory of 3D point reconstruction [17], relative point accuracy and stability decreases with the distance of the target to the cameras as the intersection angle for 3D point reconstruction becomes smaller. For measuring distances higher than approx. 100 m the low cm-level is exceeded in the stability results, leading to unreliable point measurements as well as increased system jitter. For $d_{base}\approx 9$ m, measurements above 100 m could not be performed due to immobile objects that were in the line of sight of *Camera 1*.

As shown in Table 5, similar stability results were found for $d_{base}\approx 3$ m throughout the volume. Since no objects were in the line of sight, target identification and tracking could be obtained until 120 m distance. However, we observed instabilities in the calibration process leading to unreliable point measurements for $d_{base}\approx 3$ m and higher point accuracy deviations compared to the previous experiments for $d_{base}\approx 9$ m. This was found due to a insufficient blob coverage of only about 50% in both camera images in the specific test environment. Since we could not repeat the field test, we further investigated this issue, as described in Section 5.

**Table 5:** Empirical standard deviation ${\hat{\sigma }}_{C}$ for $d_{base}\approx 3$ m.

$d_{\mathrm{track}}$ (m)	${\hat{\sigma }}_{x}$ (mm)	${\hat{\sigma }}_{y}$ (mm)	${\hat{\sigma }}_{z}$(mm)
20	0.03	0.03	0.15
30	0.13	0.10	1.74
40	0.14	0.08	2.22
50	0.32	0.17	4.57
60	0.39	0.14	4.08
70	0.72	0.15	5.90
80	2.79	0.41	7.47
90	2.97	0.54	10.72
100	2.71	0.43	18.21
110	6.31	0.85	26.77
120	4.24	0.82	31.60

To summarize our findings of the overall tracking performance, the target prototype has a maximum measuring range of approx. 120 m under good conditions (clear atmosphere, good visibility, rectangular viewing direction of the IR-LEDs towards the camera). At greater distances, the IR-LEDs cannot be reliably segmented by the *Model Identification* pipeline anymore. Up to 80 m, the points’ stability is reliable, resulting in robust point measurements and small system jitter. Increasing the distance between the LEDs associated with a higher radiant intensity of each LED would provide an improved target visibility and tracking stability at larger distances.

##### Influence of Vibrations

To evaluate the influence of heavy vibrations, such as the wheel loader engine, to relative point accuracy and stability, the targets were measured in 20,40,60 m distances during standstill with engine shutoff (*Test 1*) and at standstill while the machine’s motor was running (*Test 2*). For each run at each distance, about 200 frames were evaluated with 10 fps. The comparison of the tracking results of the horizontal wheel loader target is described in Table 6.

**Table 6:** Comparison of relative point accuracy $x_{RMS}\left(P\right)$ and standard deviation $\hat{\sigma }\;\left(C\right)$ without (motor shut off) and under heavy vibrations (motor running).

$d_{\mathrm{track}}$(m)	Test 1				Test 2			
	${\hat{x}}_{\mathrm{RMS}}\left(P\right)$(mm)	${\hat{\sigma }}_{x}$(mm)	${\hat{\sigma }}_{y}$(mm)	${\hat{\sigma }}_{z}$(mm)	$x_{\mathrm{RMS}}\left(P\right)$(mm)	${\hat{\sigma }}_{x}$(mm)	${\hat{\sigma }}_{y}$(mm)	${\hat{\sigma }}_{z}$(mm)
20	7.36	0.10	0.09	0.33	7.37	0.68	0.42	2.30
40	32.63	0.17	0.16	0.68	32.51	0.54	0.39	3.40
60	53.40	0.81	0.41	3.52	53.23	1.07	0.47	4.61

Since no predictive filtering was applied during evaluation, the table shows the unaltered results of the influence of external vibrations. The tests reveal that the system’s jitter increase from sub-millimeter to low millimeter deviation when the wheel loader’s engine is running. However, the increased jitter was not found to be strong enough to have a significant influence on relative point accuracy. This is due to the fast shutter speed of 1000 μs which should be further decreased to account for this very fast movements of the target; thereby, standard deviation could be reduced as well.

#### 4.4.6. Tracking Performance for Machine Guidance

As described in Section 4.4.2, a wheel loader was equipped with the two line targets (Figure 30b) and tracked during operation to gain practical experience in the performance and capability of the system prototype for machine guidance applications. Currently the system provides ten 3D position estimates per second (10 fps). For mining and tunneling applications such as machine guidance, this update rate is already sufficient.

**Figure 31:**
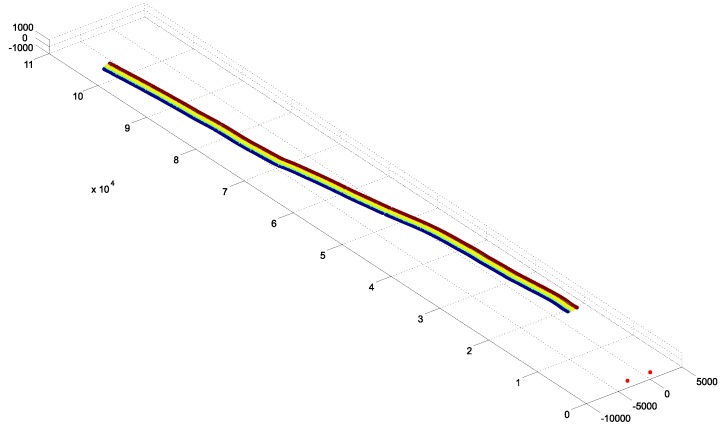
Kinematic tracking of the horizontal target from 20–110 m with $d_{base}\approx 3$ m.
- Tracking with Occlusions and Poor Visibility: Next, disturbing infrared light sources were held in the line of sight and fog has been produced artificially by a machine to simulate difficult environmental conditions and other disturbing influences. In the following, three example images are given to test both environmental interferences. In each example, both targets are simultaneously tracked and the data output of the tracking pipeline indicates a successful target tracking of the horizontal target with blue crosses, and green for the vertical target. In case of occlusions, a successful target model identification is marked by yellow crosses.
  To test the system’s robustness to filter interfering lights, both targets were tracked over a sequence of 499 subsequent frames while the wheel loader was positioned at a distance $d_{track}=23$ m in front of the cameras with $d_{base}\approx 9$ m. The horizontal target could be tracked in each frame of the sequence as it was not heavily affected by the interfering lights. The vertical model was subject to heavy occlusions and interferences. It’s model could be fully identified in more than 50% of the frames. In 206 frames, occlusions of one or more IR-LEDs occurred. In case of one occluded LED, the target model could still be identified and its 3D epicenter was estimated after occlusion recovery was performed. In the accuracy and stability data, we found comparable results to the measurements obtained during accuracy and stability evaluation in Section 4.4.5 (see Table 4) with $x_{RMS}\left(P\right)=8.90$ mm and ${\hat{\sigma }}_{x/y/z}=0.09/0.11/0.32$ mm. This demonstrates the robustness of model identification and recovery of the tracking pipeline.
  As it can be seen in Figure 32 and Figure 33, the manually inserted disturbing lights only affect the model identification in case that the interfering lights are directly in front of or very close to the target’s IR-LEDs. This case is given in Figure 32 where the heavy interference leads to the occlusion of one LED of the vertical target. However, the tracking pipeline is still able to correctly identify the target model, as indicated by the yellow crosses in Figure 32b. Thereby, the system is able to subsequently recover the missing LED in 3D for epicenter estimation. The heavy light interference that is illustrated in Figure 33 did not lead to occlusions of the target due to the LED’s properties. Both targets’ models are fully identified and tracked by the tracking pipeline despite the interference.

- Tracking under Normal Visibility: First, we tracked the wheel loader under normal visibility conditions during driving operation from 20–110 m. The estimated 3D points of the target are depicted in Figure 31 where only the horizontal target is shown for better visualization. The wheel loader was tracked over a sequence of 2560 frames; in only two frames of this data set tracking was not successful. All tracking results are directly plotted, as no filtering to remove outliers is applied. Thereby, the robustness and accuracy of the entire tracking pipeline could be objectivity evaluated, resulting in a robust and continuous tracking.

**Figure 32:**
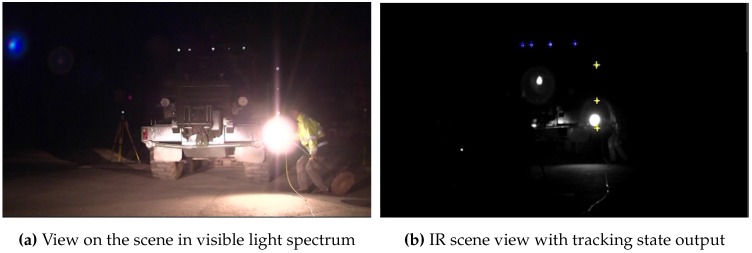
The vertical target is successfully identified (yellow crosses) despite occlusions.

**Figure 33:**
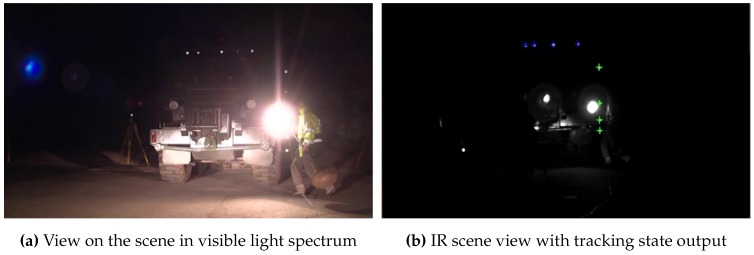
Both targets’ models are identified and tracked despite heavy interfering light.

**Figure 34:**
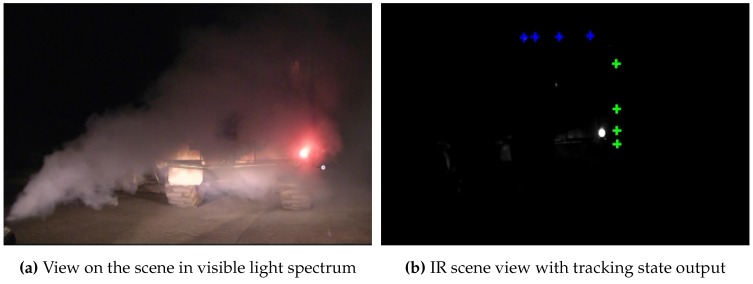
Both targets’ models are fully identified and tracked during fog tests.
  Poor visibility due to fog or dust clearly reduces the measuring range and the tracking update rate. This is a common disadvantage of all optical tracking systems as well as geodetic total stations. However, as it is depicted in Figure 34, our system is able to cope even with dense fog in front of the IR-LEDs since their radiant intensity is strong enough. Tracking loss was only temporary for a few frames and system readiness was immediately and automatically reestablished as soon as visibility improves. This is a huge advantage compared to *i.e.*, geodetic total stations, where tracking loss requires additional sighting of the target.

## 5. Discussion

We have experimentally evaluated the tracking system’s performance properties in three different wide area tracking environments, all featuring unconstrained lightening conditions and two having additionally harsh characteristics. The proposed system provides quick setup since it needs a minimal hardware setup consisting of two high quality machine vision cameras and a standard (portable) workstation for data processing. Besides stereo camera setup, pre-conditioning of the tracking volume is not required since interfering lights during camera calibration and tracking are filtered out and partly occluded targets can be recovered. Targets are designed to be re-configurable and are equipped with standard infrared light emitting diodes. We demonstrated the system’s capabilities to extrinsically calibrate the stereo camera system as well as target tracking despite heavy interferences (lights, fog). Thus, the tracking system can operate during on-going activities in the volume, featuring it to be highly unobtrusive. The system offers tracking at interactive frame rates with centimeter precision of the relative 3D position estimates up to 100 m.

1. We proposed a wide area tracking prototype that can be used for user tracking in VR/AR applications. Our results demonstrate relative 3D point accuracy $x_{RMS}\left(P\right)\;<\;9.2$ mm with sub-millimeter static position jitter $\hat{\sigma }=0.0675$ mm throughout the entire tracking volume, ranging from 5–30 m. We tested our system with several different target constellations, which can be detected within both camera views with rotations yaw and pitch from $0^{\circ }$–$45^{\circ }$ as well as roll from $0^{\circ }$–$360^{\circ }$. To our best knowledge, no competing approach provides comparable accuracy for this range, especially not with the minimal amount of only two cameras. Therefore, the presented system goes clearly beyond state-of-the-art.
2. We demonstrated the capabilities of optical tracking to be applicable to measurement scenarios beyond virtual reality environments. By providing robust hardware encasement and a simple but flexible target design, it can be used in underground scenarios such as tunnels and mines. It can be simultaneously used for a large variety of independent underground surveying tasks, such as setting out, profile control, deformation monitoring, personnel tracking for safety and machine tracking. It provides relative 3D point accuracy with a deviation of ≤21.98 mm throughout the tracking volume of 12 m× 8 m × (30–70) m. Up to 80 m, we demonstrated relative point accuracy of $x_{RMS}\left(P\right)\;<\;60.72$ mm with a very high distance-invariant stability, indicated by the (sub)-millimeter static position jitter (${\hat{\sigma }}_{x}=1.05$ mm, ${\hat{\sigma }}_{y}=0.59$ mm, ${\hat{\sigma }}_{z}=4.71$ mm). Compared to state-of-the-art underground measurement systems, our approach has the capabilities of (1) automatically starting to track one or multiple targets as soon as the target is within the view of the vision system, thus manual sighting can be omitted; (2) tracking moving as well as partly occluded targets; (3) provides a flexible target design that allows general usage of various tracking and measuring tasks and (4) addresses the need for highly automated positioning systems [33,69].

### 5.1. Position Estimation

Compared to indoor tracking technologies, such as RFID that support multiple targets in a large volume, our proposed system supersedes pre-conditioning of the tracking volume to provide cost- and time-efficiency. Comparing the presented system to state-of-the-art infrared optical tracking systems in terms of range coverage and accuracy, it significantly extends the available tracking range up to 100 m while requiring only two cameras and providing a relative 3D point accuracy with sub-centimeter deviation up to 30 m and low-centimeter deviation up to 100 m, as shown in Table 1, Table 2 and Table 4. To our best knowledge, none of the existing systems, as described in Section 1.2.2 gives accuracy specifications for distances greater than 10 m. Due to the implicit line characteristic of the target design, orientation can only be provided up to two DOFs. However, for user head tracking, this can be compensated by combining several line targets into one composite target. Tracking accuracy in terms of orientation has not been part of this work and will be evaluated in the future. For underground surveying tasks, the achieved relative 3D point accuracy is adequate for machine guidance but was found not accurate enough for tasks such as setting out. However, the following aspects were identified to increase the accuracy. Extending the baseline results in better depth accuracy, while using an image sensor with higher resolution minimizes segmentation inaccuracies that leads as well to enhanced precision. The main aspect of optimization was found in the extrinsic calibration approach.

In addition, the following aspects have been identified during evaluation to further optimize the system. (1) The software prototype of the proposed tracking pipeline offers interactive frame rates. However, the MATLAB image processing components [70] should be replaced by C/C++ modules and parallelization should be exploited to decrease tracking latency. This reduces this shortcoming to a pure software development task; (2) As every optical technology, the proposed system requires good visibility. In presence of strong fog and dust, the achievable measuring range is reduced, however, this effect can be partly mitigated by using LEDs with higher radiant intensity as well as LED arrays.

### 5.2. Calibration

The evaluation of our proposed calibration method indicates promising results. Despite interfering lights, the target’s LEDs are robustly segmented to ensure sufficient and reliable camera parameter estimation. However, tests revealed some limitations of the current approach. The manual movement of the target through the volume keeps the tracking system independent from additional (fixed-installed) visual features. However, not all areas of the camera image can be covered and most blobs are found in the camera images’ center which results in an unbalanced blob distribution, as depicted in Figure 20 and Figure 26. Especially in the vertical direction, distribution is limited by human size and the length of the calibration target as well as by the natural boundaries of the physical environment, such as the ceiling and the ground. The distribution can be improved by using a longer calibration apparatus but as stated only to a certain extend. Therefore, a future aspect of the research is to use additional visual features that are extracted from the environment and fuse them with the blob features to increase the feature distribution along the edges and in the corners of the images. In a well illuminated environment, *i.e.*, for VR/AR tracking, natural features can be extracted from the environment. In an underground environment, where illumination is poor and geometric structures are mostly found around the front face, natural feature extraction would not significantly enhance the feature distribution in the camera images. Here, the installation of additional single IR-LED markers would serve as an adequate solution. They could be equally distributed within the tracking volume and autonomously detected and subsequently extracted by exploiting the radio connection to remotely control the LEDs state. Thereby, the system’s unique features to function in an unconstrained environment while requiring a small amount of hardware and little user interaction would be retained. The author has conducted research and initial tests with the proposed solution, the work has not been published until the submission of this work.

## 6. Conclusions

In this paper, a robust wide area optical tracking approach was presented that estimates the 3D position of model-based targets across three different use cases. The approach extends state-of-the-art optical tracking systems by proposing a robust extrinsic stereo camera calibration, by presenting a highly re-configurable target design, and by providing a software-based processing pipeline that enables the system to cope with large tracking distances, static and moving interfering lights, partly occluded targets as well as disturbances such as fog and dust during calibration and tracking. We employ projective invariant property matching to robustly identify the model-based optical apparatus (target) that is used for extrinsic calibration and tracking. For estimating the external camera parameters, the apparatus is used to artificially generate 0D image features that are crucial in poorly illuminated environments with little geometric structure. Furthermore, the target’s properties support reliable correspondence matching without requiring the epipolar geometry for correspondence analysis. During tracking, the approach allows model fitting already in the 2D image domain that results in a drastically reduced set of correspondence candidates. This in turn considerably decreases the combinatorial complexity of the multiple-view correlation problem.

Summarizing, the demonstrated system’s properties allows for robust and cost efficient wide area position tracking that is required by a number of indoor application areas that require spatial context awareness, such as navigation, automated surveying, VR/AR, entertainment as well as remote object control. By overcoming limitations of existing optical systems, it can foster the further emerging of the aforementioned applications areas within wide indoor environments that are currently impeded by the limitations of state-of-the-art systems.

### Future Directions

The evaluation of the entire system across the three use cases revealed the following open topics to be addressed in future research.

- To enhance the estimation of external camera parameters in terms of robustness and accuracy, feature distribution in the camera image should be improved. We found an unbalanced blob coverage of the artificially generated point features especially in the vertical dimension that is caused by limited human size and the length of the calibration target as well as by the natural boundaries of the physical environment, such as the ceiling and the ground. Therefore, we will investigate concepts to extract natural features from distinct environmental structures and fuse them with the blob features to increase the distribution along the edges and in the corner of the images. This approach requires a well illuminated environment with a sufficient amount of prominent geometrical structure that might be given in a standard indoor environment. In an underground scenario, where illumination is poor and geometric structures are mostly found around the front face, natural feature extraction would not significantly enhance the feature distribution in the camera images. Here, additional single IR-LED markers that are installed throughout the volume would be an adequate solution to improve the feature distribution. These single blob features could be autonomously detected and extracted using the proposed hardware interference filtering approaches from Section 2. With these methods, we hope to achieve a more accurate calibration for stereo rigs with large baseline in both illuminated as well as poorly illuminated and non-cluttered environments.
- To extend the field of view and thereby, the horizontal and vertical tracking coverage, the relative point accuracy should be evaluated with different hardware setups using higher resolution cameras and lenses with smaller focal length. Additionally, we will examine infrared LEDs with less radiant intensity to reduce the tracking target length. Both aspects can be beneficial especially for tracking at smaller distances up to 30 m.
- To obtain absolute 3D coordinates for surveying measurement tasks, linking the camera’s coordinate system to the geo-reference coordinate system is required. The geo-reference coordinate system is obtained by geodesic measurements using a total station/theodolite. To determine the transformation matrix between the two coordinate systems, we plan to equip the tracking targets as well as additional stationary single point targets with geodesic prisms that are measured with a theodolite to obtain highly accurate geo-referenced 3D measurements.
